# The Impact of Maternal Obesity and Diabetes on the Development of Congenital Heart Defects (CHDs) in Offspring: A Narrative Review

**DOI:** 10.3390/metabo16050341

**Published:** 2026-05-19

**Authors:** Marek Zubrzycki, Mariusz Kuśmierczyk, Jan Fritz Gummert, Angelika Costard-Jäckle, Lech Paluszkiewicz, Tobias Hecht, Ingvild Birschmann, Anna Zubrzycka, Maria Zubrzycka

**Affiliations:** 1 Department of Surgery for Congenital Heart Defects, Heart and Diabetes Center NRW, University Hospital, Ruhr-University Bochum, Georgstr. 11, 32545 Bad Oeynhausen, Germany; m.zubrzycki@outlook.com; 2 Department of Cardiothoracic Surgery and Transplantology, Medical University of Warsaw, 02-097 Warsaw, Poland; mariusz.kusmierczyk@wum.edu.pl; 3 Clinic for Thoracic and Cardiovascular Surgery, Heart and Diabetes Center NRW, University Hospital, Ruhr-University Bochum, Georgstr. 11, 32545 Bad Oeynhausen, Germany; jgummert@hdz-nrw.de (J.F.G.); ajaeckle@hdz-nrw.de (A.C.-J.); lpaluszkiewicz@hdz-nrw.de (L.P.); 4 Department of Congenital Heart Disease/Pediatric Cardioloy, Heart and Diabetes Center NRW, University Hospital, Ruhr-University Bochum, Georgstr. 11, 32545 Bad Oeynhausen, Germany; thecht@hdz-nrw.de; 5 Heart and Diabetes Center of Nordrhein-Westfalen, Institute of Laboratory and Transfusion Medicine, University Hospitals, Ruhr-University Bochum, Georgstr. 11, 32545 Bad Oeynhausen, Germany; ibirschmann@hdz-nrw.de; 6 Clinic of Gynecology, Fertility, and Obstetrics Salve Medica, Szparagowa 10, 91-211 Lodz, Poland; azubrzycka@op.pl; 7 Department of Clinical Physiology, Faculty of Medicine, Medical University of Lodz, Mazowiecka 6/8, 92-215 Lodz, Poland

**Keywords:** metabolic syndrome, maternal obesity, maternal diabetes, cardiac development, congenital heart defects, genetic, epigenetic and environmental factors

## Abstract

Congenital heart disease (CHD) is the most common anatomical malformation occurring in live-born infants and an increasing cause of morbidity and mortality all over the world. Population-based observations have described associations between maternal cardiometabolic disorders and the risk of CHD in offspring. The present article is a narrative review. The aim of this study was to review the epidemiological evidence and clinical observations relating maternal obesity and diabetes mellitus to the risk of CHD in offspring, with particular attention paid to first trimester disturbances of fetal cardiac development and the influence of genetic, epigenetic and environmental factors. Studies have shown that maternal diabetes is a risk factor associated with nearly all subtypes of CHDs in offspring, while obesity and overweight are associated with increased risk for complex defects and outflow tract obstruction and decreased risk for ventricular septal defects. Diabetes and obesity share several phenotypes, which could be transmissible from mother to fetus via the placenta. This means that an increase in maternal glucose could be responsible for the prevalence of CHD in newborns of obese women. On the other hand, maternal diabetes may induce epigenetic modifications in the developing fetus. DNA methylation changes can impact gene expression patterns relevant to heart development. The abovementioned studies are heterogenous, express different opinions and are often difficult to compare. Therefore, the results from these meta-analyses must be interpreted with caution. Optimal diabetes control is responsible for the prevention of oxidative stress in diabetic pregnancies, and a deeper understanding of maternal risk factors holds the potential to improve both prenatal detection of CHDs by identifying at-risk pregnancies and primary prevention of diseases by improving preconception management.

## 1. Introduction

Congenital heart disease (CHD) arises from anomalies in the heart and large blood vessel formation in the course of embryonic development. The prevalence of CHD affects ≈1% of live births and accounts for 30% of fetal deaths [1,2]. The spectrum of CHDs varies. They can be diagnosed as an isolated finding or as part of a collection of findings. The etiology of CHDs is complex and multifactorial, with 80% of cases attributed to the interplay between genetic and environmental factors [3,4,5]. Maternal environmental risk factors, such as gestational diabetes mellitus, obesity, maternal age, drug use, air pollution, smoking, alcohol consumption, febrile illnesses during pregnancy, viral infections, pregnancy through artificial reproductive technologies, and socioeconomic factors, have been linked to CHD development in offspring [3,4,6,7,8,9].

Among genetic factors, single-gene disorders, chromosomal anomalies, and double-gene disorders can be distinguished [5,10,11]. The key genes involved in transcriptional control, signaling and morphogenesis have been identified thanks to advances in understanding of the molecular mechanisms of heart development. Some of these genes are involved in the development of CHDs. The most common genes or genetic loci associated with CHD anomalies include *NKX2-5*, *GATA4*, *TBX5*, *SRD5A2*, *MTHFR*, *MTRR* and *MTR*, *CFTR*, and 1p22 and 20q12 anomalies [3,5,10,11,12]. Most genetic CHDs exhibit an autosomal dominant inheritance pattern, but several investigations have led to the identification of non-Mendelian factors that contribute to CHD [3,5,10,11]. In addition to genetics, epigenetics plays a crucial role in heart development, with mounting evidence suggesting its dysregulation in CHD pathogenesis [12,13,14]. Both high and low miRNA expression levels have been shown to cause CHDs [15,16,17,18].

Among environmental contributors, metabolic diseases such as obesity, maternal pregestational diabetes mellitus (PGDM) and gestational diabetes (GDM) are highly prevalent and well-established risk factors for CHDs in offspring worldwide, especially in industrialized countries [4,19,20,21,22,23,24,25]. Although maternal factors are recognized risk factors for CHDs, the underlying molecular mechanisms still remain unclear [19,26,27]. The link between obesity and diabetes is thought to be mediated by chronic inflammation, oxidative stress, and dysregulated angiogenic signaling, which may contribute to endothelial dysfunction and abnormal placental development, which in turn is implicated to the pathogenesis of CHDs [28,29].

Several meta-analyses conducted by different research groups have consistently identified that the increased risk associated with maternal obesity includes a wide range of different heart defects, including transposition of the great arteries (TGA); tetralogy of Fallot (ToF); atrial, ventricular and atrioventricular septal defects (ASDs, VSDs, and AVSDs); aortic arch defects; persistent ductus arteriosus (PDA); left ventricular outflow tract obstruction defects (LVOTOs) and right ventricular outflow tract defects (RVOTOs); and univentricular heart (UVH) [21,30,31,32]. Although some studies have reported no increased risk for conotruncal defects (CTDs), other studies reported risk elevations for TGA, as well as defects of the great vessels and truncus arteriosus [33,34,35,36].

Also, meta-analyses carried out by several groups have identified the increased likelihood of specific CHD phenotypes upon exposure to maternal hyperglycemia. Maternal pregestational diabetes mellitus (PGDM) was associated with a higher risk of TGA, persistent truncus arteriosus (PTA), heterotaxy, VSDs and ASDs, ToF, double-outlet right ventricle (DORV) and hypoplastic left heart syndrome (HLHS) in offspring [23,37,38,39,40].

Maternal gestational diabetes mellitus (GDM) has been associated with higher risks of ASDs, VSDs, and ToF [41]. The study by Chen et al. [42] suggested that maternal diabetes mellitus (matDM) was significantly associated with most phenotypes of CHDs, particularly double outlet of the right ventricle, atrioventricular septal defect and truncus arteriosus. Furthermore, this study suggests a significantly higher risk of CHDs among mothers with PGDM than in those with GDM [42].

Understanding the molecular mechanisms of induction of CHDs by a number of environmental and genetic factors and identifying the stages of heart formation that are most vulnerable to these factors will aid the design of epidemiological studies to assess the extent of these risks in human CHDs. A prerequisite for prevention of CHDs is a better understanding of potential modifiable risk factors. Therefore, primary prevention of overweight and obesity in women of reproductive age and careful treatment of PGDM may hold the opportunity to reduce the burden of CHDs.

## 2. Methodology

The present article is a narrative review. In this review, we studied the factors associated with maternal obesity and GDM. We also discussed the potential underlying mechanisms of the observed associations and direct complications in the fetus/offspring in the form of CHDs. This work is a compendium of knowledge derived from clinical and genetic studies in humans and animal models. It is based on the latest publications and available data on the occurrence of CHDs in the offspring of mothers with diabetes and obesity.

A comprehensive literature search in this narrative review was conducted using the following inclusion criteria: (a) publications in English describing the impact of obesity and the impact of diabetes in mothers on the development of CHDs in their offspring; (b) observational studies involving women and experimental studies involving animals; (c) laboratory tests with assessment of obesity—body mass index (BMI)—and assessment of diabetes—fasting blood glucose, oral glucose tolerance test (OGTT), and glycated hemoglobin A (HbA1c); (d) patients with confirmed diagnosis of CHDs; (e) clinical studies. All possible articles, whether original studies or reviews, i.e., cohort studies, retrospective studies, prospective studies and meta-analyses, were considered for this review. The review also provides an overview of the current understanding of the influence of genetic, epigenetic and environmental factors on the cardiac development and the development of CHDs in the offspring of obese and diabetic mothers. The final search included the most valuable and highest-rated peer-reviewed articles published in the last two decades until the end of 2025, all of which are available through the Pub-Med/Medline/EMBASE/Cochrane Library database. The databases were searched using the following keywords: “metabolic syndrome”, “maternal obesity”, “maternal diabetes”, “cardiac development”, “congenital heart defects”, “genetic factors”, “epigenetic factors”, and “environmental factors”. Additional records were identified using cross-references. A manual search was also conducted combining “AND” or “OR” operators. All identified problems were described in the order of occurrence of CHDs in the offspring of diabetic and obese mothers on different continents of the world and the key mechanisms influencing the development of CHDs in the offspring of diabetic and obese mothers were described.

The following exclusion criteria were applied: (a) unpublished articles or conference proceedings; (b) editorials, opinions, case reports, and letters to the editor; (c) studies in which the patient’s diagnosis is uncertain; (d) abstracts.

This review has been conceptualized to address the need for an up-to-date combined review of the mechanisms of CHD development in the fetuses/offspring of mothers with diabetes and obesity aiming to include all the new data in a simple and comprehensive manner.

## 3. Metabolic Syndrome (MetS)—Definition and Diagnostic Criteria

In 1988, Reaven described metabolic syndrome (MetS), initially known as syndrome X, for the first time [43,44]. The most common definition of MetS includes a co-occurrence of abdominal obesity and at least two cardiometabolic risk factors such as hypertension, insulin resistance (IR), and dyslipidemia, as well as a low concentration of high-density cholesterol (HDL) (Table 1 and Table 2) [45,46,47,48].

A controversy regarding the definition of MetS and its usefulness has arisen recently. Different clinical criteria for the definition of MetS have been adopted by international organizations such as the International Diabetes Federation (IDF) and the World Health Organization (WHO).

Table 2 summarizes three of the most commonly used definitions of metabolic syndrome [49].

Their criteria are similar in many aspects, but they also reveal fundamental differences in their positioning of the predominant causes of the syndrome. All groups agree on the core components of metabolic syndrome: obesity, insulin resistance, dyslipidemia, and hypertension. These conditions are interrelated and share the underlying mediators, mechanisms and signaling pathways.

However, the pathophysiology of metabolic syndrome is not fully understood. Environmental and genetic factors are considered to play an important role in the development of MetS. The genes involved in the development of MetS include genes influencing insulin sensitivity (encoding PPAR-γ and CAPN10), lipid metabolism genes (encoding CDS36 and 11-β-HSD), genes regulating free fatty acid metabolism (encoding adiponectin and β-adrenergic receptor), monogenic obesity genes (leptin genes) and genes associated with inflammatory conditions (TNF-α and CRP genes) [50,51]. Patients with MetS have increased levels of inflammatory markers, including C-reactive protein, TNF-α, interleukin 6 (IL-6), and fibrinogen. In addition to inflammation, oxidative stress is also attributed to play some role in the genesis of MetS [48]. During the reproductive period, the first manifestation of metabolic syndrome in women may be the onset of gestational diabetes or preeclampsia [49,52].

MetS is an increasingly common disorder affecting thousands of people worldwide, especially in industrialized countries. Its average prevalence reaches 30% in adults [53] and between 6 and 39% in children/teenagers [54]. Approximately 24% of adults in the USA, 12–37% of the Asian population and 12–26% of the European population suffer from this disease [55]. With the improvement in living standards and an increasing number of women of advanced maternal age, there is an increase in the prevalence of chronic metabolic diseases, such as obesity and diabetes, among pregnant women [4,56,57]. MetS was assessed in pregnant women (5628) in a multicenter study from Australia, New Zealand, Ireland and the United Kingdom and was reported to occur with an incidence of 12.3% at 15 weeks’ gestation [58]. An association between increased risk of CHDs and maternal metabolic disorders such as obesity [30,31], diabetes [23,38,59], hypertension [39,60], and preeclampsia (PE) [61,62] was reported by large cohort studies from Scandinavia and North America. All the aforementioned metabolic disorders can be associated with hyperglycemia and underlying insulin resistance, and thereby with MetS [46,63].

MetS increases the risk of cardiovascular complications occurrence 2-fold and even up to 5-fold in the case of type 2 diabetes mellitus (T2DM) [64]. It is possible that combinations of several maternal metabolic disorders in the same pregnancy are likely to be associated with higher risk of CHDs in offspring as compared to women with a single disorder [21]. Women with MetS are at increased risk of complications during pregnancy. The most common include miscarriage, gestational diabetes, hypertension, and thromboembolic events.

## 4. Cardiac Development

Prior to delineation of the complex mechanisms underlying the etiology of congenital heart defects (CHDs) associated with metabolic syndrome diseases, it is essentially important to understand the normal heart development and the cellular and molecular pathways that regulate its development [65,66,67]. Their disruption in each step of organogenesis by genetic, epigenetic and environmental factors acting on the mother may lead to the occurrence of CHDs in the offspring [3,4,8,9]. A possible impact of maternal metabolic status, including diabetes and obesity, on both the morphological and functional development of the offspring heart has been demonstrated by several large-scale cohort studies [68,69].

Cardiac development is a highly complex process involving the interplay of several cell lineages and cellular processes, all tightly controlled by their corresponding genetic programs [65].

The heart is one of the earliest organs to develop in the course of embryonic development [67]. During cardiogenesis, differentiating precursor cell clusters interact to generate specialized heart cells with a well-defined three-dimensional architecture in the restricted regions. The process starts at approximately 15–19 days of human pregnancy from the specification of cardiac progenitor cells [9,70,71]. First, mesodermal cells constitute a heart-forming region known as the cardiac crescent, which contains myocardial precursor cells. The precursor cells are classified as either first heart field (FHF) or second heart field (SHF) cells. The FHF cells ultimately become the heart tube, which then forms the definitive left ventricle and atrioventricular canal. Growth of the early heart tube occurs by the progressive addition of SHF progenitor cells to the cardiac poles. The SHF gives rise to ventricular septal, right ventricular and outflow tract (OFT) myocardium at the arterial pole, and atrial, including atrial septal, myocardium, at the venous pole. SHF deployment creates the template for subsequent cardiac septation and has been implicated in cardiac looping and in orchestrating outflow tract development with neural crest cells [66,72].

As SHF progenitors incorporate, the heart tube elongates, buckles and loops to the right from around 20 to 25 days of human development to ensure the proper cardiac segment alignment template, in preparation for chamber septation [72]. An additional source of the formation of cardiac structures is the “heart pool” of neural crest cells, which are not of mesodermal origin but are derived from the neuroectoderm. The cardiac neural crest cells, which migrate towards the primary heart tube, are involved in the formation of the arteries of the pharyngeal arches, the outflow tract of the right ventricle, the cardiac ganglia, and, together with the pre-epicardial organ, the conduction system of the heart [9,72].

Atrial and ventricular septation begins at 31–35 days of human pregnancy. At that stage, differentiation of the ventriculoarterial and atrioventricular valve systems, including the tendinous cords and papillary muscles, occurs. Concurrently, the conduction system and coronary vessels are developed [73].

**Figure 1:**
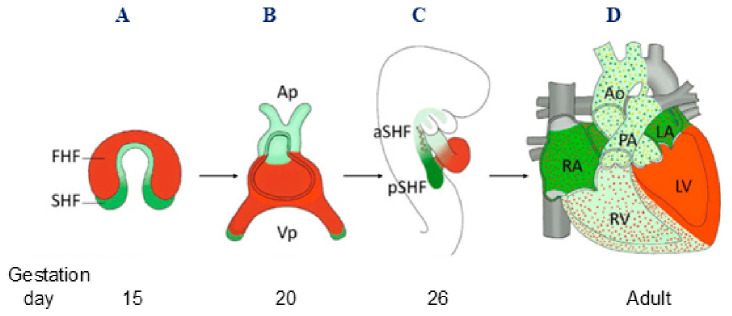
Scheme of the main stages of human cardiac development. Schematic representation of the formation of a heart loop: (**A**) cardiac crescent configuration; (**B**) the cardiac crescent is transformed into a transiently linear heart tube; (**C**) rightward looping; (**D**) the mature four-chamber heart is formed. Abbreviations: FHF: first heart field; SHF: second heart field; RV: right ventricle; LV: left ventricle; RA: right atrium; LA: left atrium; Ap: arterial pole; Vp: venous pole; PA: pulmonary artery; Ao: aorta. This figure has been taken from the article of Ibrahim et al. [8] distributed under the terms and conditions of the Creative Commons Attribution License (CC BY-NC-ND/4.0).

During days 37–44, the development of the heart tube is completed and the basic structure of the four heart chambers is formed (Figure 1) [8,74,75,76,77]. We described the development of the heart in detail in Part I of the review by Zubrzycki et al. [9].

Heart development relies on crucial spatiotemporal interactions between distinct multipotent cardiac progenitors during early embryogenesis. Precise regulation of gene expression is crucial for correct cardiogenesis and cardiac differentiation. A highly coordinated signaling network of NODAL, NOTCH, BMP, WNT, HIF1α, TGFβ and FGF induces expression of a core group of cardiac transcription factors, including NKX2–5, GATA4/5/6, HAND1/2, MEF2, TBX1/5/20, and ISL1, that function in a mutually reinforcing cascade to drive lineage restriction and differentiation of progenitor cell populations (i.e., FHF and SHF) to chamber-specific cardiac cell types [77,78,79]. Any dysregulation of these pathways during embryonic development may disrupt the spatiotemporal regulation of complex three-dimensional heart structures, leading to CHDs in the fetus. The severity of the CHD phenotype depends on the timing and type of dysregulation.

## 5. Classification of Congenital Heart Defects (CHDs)

Congenital heart defects (CHDs) are anatomical abnormalities caused by disruption of normal development of the heart and/or large blood vessels during embryonic development [80]. CHDs are the most prevalent type of birth defect worldwide and are the leading cause of death for the fetus and in the first year of life [81,82,83]. Although advances in clinical management have improved the survival of children with CHDs, adult survivors commonly experience cardiac and non-cardiac comorbidities, which affect their quality of life and prognosis [84,85,86].

CHDs account for 0.9–1% of all live births and 10% of stillbirths [2,81,82,87,88,89,90,91]. The prevalence of CHDs is increasing worldwide, most probably with the rising incidence of metabolic disorders, including diabetes and obesity [56,81,88].

**Figure 2:**
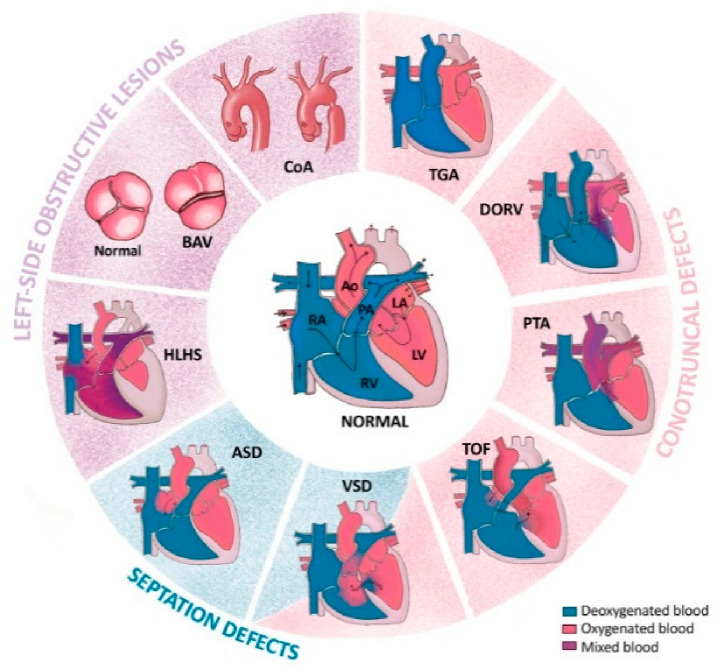
Schematic representation of the normal four-chambered heart and the structural abnormalities of the most common forms of congenital heart defects (CHDs). Abbreviations: TGA: Transposition of the Great Arteries; DORV: Double-Outlet Right Ventricle; PTA: Persistent Truncus Arteriosus; TOF: Tetralogy of Fallot; VSD: Ventricular Septal Defect; ASD: Atrial Septal Defect; HLHS: Hypoplastic Left Heart Syndrome; BAV: Bicuspid Aortic Valve; CoA: Coarctation of the Aorta; RA: Right Atrium; RV: Right Ventricle; LA: Left Atrium; LV: Left Ventricle; PA: Pulmonary Artery; Ao: Aorta. Black arrows indicate the direction of blood flow. This figure has been taken from the article of Ibrahim et al. [8] distributed under the terms and conditions of the Creative Commons Attribution License (CC BY-NC-ND 4.0).

There are more than 20 specific types of CHDs, which can be subdivided into several categories depending on their spatiotemporal origin during embryogenesis [8,92]. The most common CHD types are summarized in Figure 2.

Firstly, there are conotruncal defects resulting from an abnormal formation of the outflow tract (OFT) of the heart. They include (1) transposition of the great arteries (TGA) [93], in which the pulmonary trunk and the aorta are inverted and connected to the left and right ventricle, respectively; (2) double-outlet right ventricle (DORV) [94], involving the two major arteries, the pulmonary artery and the aorta, connected to the right ventricle with ventricular septal defects (VSDs), i.e., when the ventricular septum fails to form correctly, commonly observed with DORV; (3) persistent truncus arteriosus (PTA), also referred to as common arterial trunk (CAT) [95], when the aorta and the pulmonary artery fail to separate into two distinct structures, so the common arterial trunk aligns over a large VSD; (4) tetralogy of Fallot (ToF) [95] is the combination of several malformations: an overriding aorta that induces VSDs, pulmonary artery stenosis, and right ventricle hypertrophy. An extreme form of ToF is pulmonary atresia with VSDs.

Secondly, there are septation defects that are found to be either isolated or associated with other forms of CHDs. They include (1) the aforementioned VSDs [95]; (2) atrial septal defects (ASDs) [96], involving incorrect formation of the septum between the two atria; and (3) atrioventricular septal defects (AVSDs) [97], in which the four chambers of the heart communicate due to failure of the formation of the atrioventricular septum.

Thirdly, there are left-side obstructive lesions, occurring due to abnormal formation of the aorta. These lesions include (1) hypoplastic left heart syndrome (HLHS) [98], where the underdeveloped left ventricle has no outlet; (2) bicuspid aortic valve (BAV) [99], the most common type of CHD, where the aortic valve has two leaflets instead of three; (3) coarctation of the aorta (CoA), a localized narrowing of the aortic lumen leading to hypertension in the upper limbs; hypertrophy of the left ventricle, if severe; and poor vascularization of the abdominal organs and lower limbs [100].

These defects arise as a result of disorders in the processes of migration, differentiation or proliferation of progenitor cells in the first weeks of fetal life. Progenitor cells are involved in this process through SHF-related disorders involving the abnormal addition of progenitor cells from SHF to the main heart tube. Abnormal migration, or premature differentiation of these cells prevents the proper alignment of the aorta and pulmonary artery in relation to the ventricles. Disorders in this process lead to arterial conotruncal defects, the right ventricle and the septa of the heart. SHF is closely associated with nerve crest cells that divide the common trunk into two separate vessels. Nerve crest cells must migrate to the heart to form septa and large vessels. Disorders of this migration result in structural defects. Septal defects (VSDs and ASDs) are caused by impaired distribution of cells from both cardiac fields (FHF and SHF) and mesenchyme. Most defects concern the membranous part of the septum, formed by the combination of tissues derived from the endocardial cushions and the conotruncal septum. Left heart defects (HLHS) are primarily associated with defects in the FHF, which is the primary scaffold for the left ventricle. Hypoplasia results from an insufficient number of cardiomyocytes formed from FHF progenitors or their premature death in fetal life [9].

The pathomechanisms underlying the development of CHD have been heavily investigated using model systems like mice, zebrafish, and human pluripotent stem cells (hPSCs). These models have revealed that CHD is a complex, often multifactorial condition driven by genetic, epigenetic, and environmental factors that disrupt the key stages of cardiac morphogenesis.

## 6. Prevalence of Congenital Heart Defects (CHDs) Worldwide

The prevalence of CHDs is increasing worldwide, most probably with the rising incidence of metabolic disorders, including diabetes and obesity, the older age of women giving birth, and greater availability of diagnostic methods [56]. It has been suggested that the incidence of CHDs in newborns changes over time and varies according to maternal race, country of residence and continent [101]. Over time, the reported total CHD birth prevalence increased substantially, from <1 per 1000 live births in 1930 to 9 per 1000 live births in recent years, which is probably related to under-reporting in the past in countries with a low socioeconomic status [2,102]. Significant geographical differences were found across the continents. The highest total CHD birth prevalence was reported in Asia (especially in Iran and India) (9.3 per 1000 live births) and the lowest in Africa (1.9 per 1000 live births) [103]. The reported total CHD birth prevalence in Asia was significantly higher than in Europe, North America, South America, Oceania and Africa. The second highest total CHD birth prevalence (8.2 per 1000 live births) was reported in Europe. It was significantly higher than in North America, South America, Oceania and Africa.

On average, the total CHD birth prevalence in China from 1980 to 2019 was 2.502 (95% CI: 2.397, 2.607) per 1000 births. It increased significantly over time, from 0.201 per 1000 births (95% CI: 0.004, 0.398) in 1980–1984 to 4.905 per 1000 births (95% CI: 4.288, 5.521) in 2015–2019 [104]. The total CHD birth prevalence has been on the rise, except for a slight decline during the period 1990–1994, with a slow rise from 1980 to 2004 and a substantial rise from 2005 to 2019 [104].

Among total CHDs, the prevalence of most common subtypes varies, but VSDs, ASDs and PDA are consistently among the most frequent ones.

**Figure 3:**
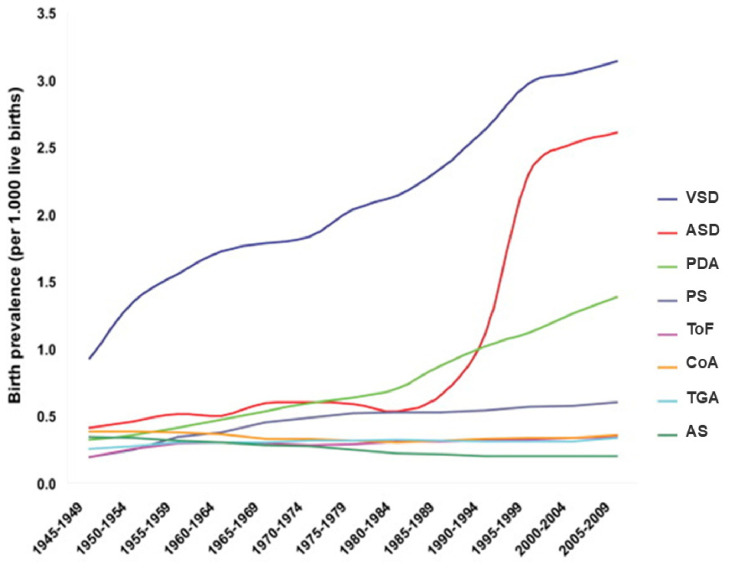
Birth prevalence of CHD subtypes over time. Time course of birth prevalence of the 8 most common CHD subtypes from 1945 to 2010. Abbreviations: AS: aortic stenosis; ASD: atrial septal defect; CoA: aortic coarctation; PDA: patent ductus arteriosus; PS: pulmonary stenosis; TGA: transposition of the great arteries; ToF: tetralogy of Fallot; VSD: ventricular septal defect. This figure has been taken from the article of Linde et al. [2] distributed under the terms and conditions of the Creative Commons Attribution License (CC BY-NC-ND 4.0).

The birth prevalence (per 1000 live births) of the eight most common CHD subtypes reported worldwide was as follows: VSDs (2.62); ASDs (1.64); PDA (0.87); pulmonary stenosis (PS) (0.50); ToF (0.34); CoA (0.34); TGA (0.31); and aortic valve stenosis (AS) (0.22) (Figure 3).

**Figure 4:**
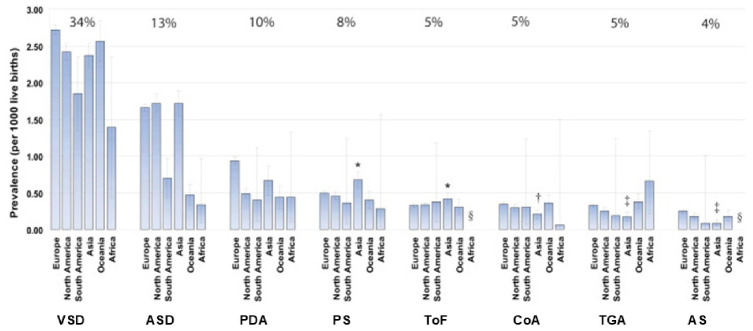
Birth prevalence of the 8 most common CHD subtypes in newborns on each continent. Distribution of subtypes among total CHDs is shown as percentages above bars. * Reported PS and ToF birth prevalence in Asia was significantly higher than in Europe (*p* < 0.001) and North America (*p* < 0.001). † Reported CoA birth prevalence in Asia was significantly lower than in Europe (*p* < 0.001). ‡ Reported TGA and AS birth prevalence in Asia was significantly lower than in Europe (*p* < 0.001), North America (*p* < 0.001) and Oceania (*p* < 0.001). § No data on ToF or AS birth prevalence in Africa were available. Abbreviations as in Figure 3. This figure has been taken from the article of Linde et al. [2] distributed under the terms and conditions of the Creative Commons Attribution License (CC BY-NC-ND 4.0).

Significant geographic differences were detected in the reported birth prevalence of the eight most common CHD subtypes (Figure 4).

It is interesting that the birth prevalence of pulmonary outflow tract obstructions (PS and ToF) is relatively high while the birth prevalence of left ventricular outflow tract obstructions (CoA and AS) is low in Asia. The above findings confirm the results of Jacobs et al. [105], who found that White children seem to have more left ventricular obstructive anomalies (LVOTOs), whereas Chinese children have more right ventricular outflow tract anomalies (RVOTOs). Furthermore, a lower birth prevalence of TGA in Asia compared with Europe, North America, South America, and Oceania (*p* < 0.001) has been reported. A possible explanation might be found in genetic origin.

In most of the studies from India, VSDs have been found to be the most common anomaly, followed by PDA. The other common anomalies are ASDs, PS and CoA. A community-based study by Bhat et al. [106] in Uttarkhand reported VSDs (30.4%) as the most common congenital heart disease followed by ASDs (17.63%), PDA (9.62%), and PS (6.41%). The burden of CHD in India is likely to be the largest among all nations in the world simply because of the fact that there are more children born in India than anywhere else [107]. In most low- and middle-income nations, including India, only a small fraction of children with heart disease can expect to receive comprehensive care in today’s times. The majority of children with CHDs in this region escape detection due to the lack of awareness, poor socioeconomic status, and poor availability of echocardiography.

## 7. Etiological Factors Contributing to CHD

The etiology of CHD is complex, involving both hereditary and non-hereditary risk factors [6,83]. Disturbances of any stage of organogenesis may lead to the occurrence of CHDs that could be initiated by various genetic, epigenetic, or environmental factors [3,4,8,9,108].

During the past several decades, a consensus has emerged that genetic factors (e.g., chromosomal abnormalities, smaller copy number variants, and point mutations) and maternal exposure to environmental factors (air pollution and toxic chemicals), parental smoking, maternal history (infectious diseases during pregnancy and pregestational and gestational diabetes mellitus), maternal obesity, maternal drug intake, and pregnancy through artificial reproductive technologies, as well as socioeconomic factors, are related to the occurrence of CHDs in the fetus [3,4,7].

**Figure 5:**
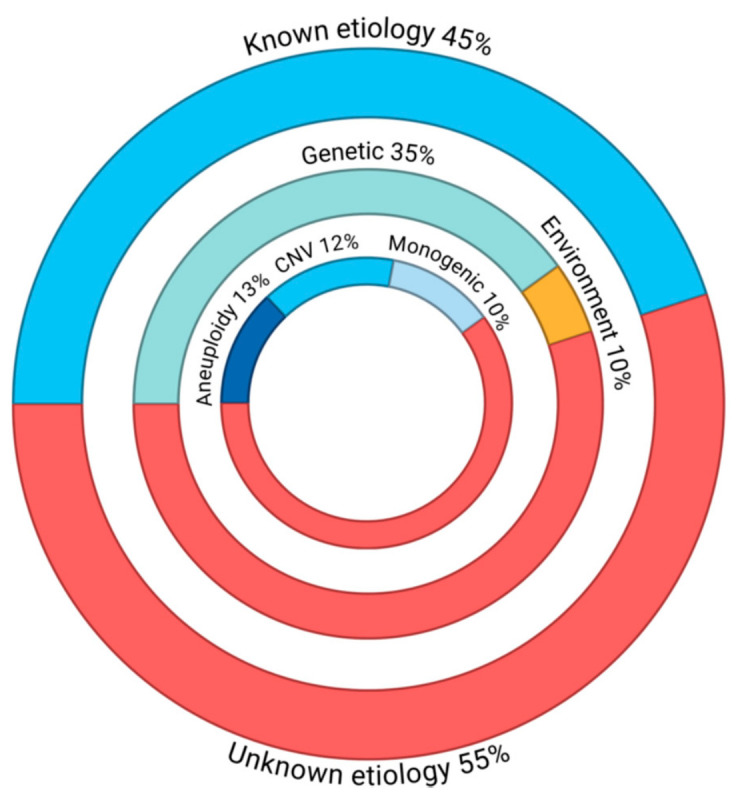
Pie chart representing the percentage of known and unknown etiologies in human CHD cases. From outside in, it shows the proportion of known vs. unknown etiologies, the proportion of genetic vs. environmental etiologies, and the proportion of known genetic etiologies and copy number variation (CNV). All percentages are approximations. This figure has been taken from the article of Choudhury et al. [3] distributed under the terms and conditions of the Creative Commons Attribution License (CC BY-NC-ND 4.0).

The breakdown of human CHD etiologies is presented in Figure 5. Approximately 45% of CHD cases can be attributed to a known etiologic factor, where ~35% are due to the presence of pathogenic genetic loci and ~10% to exposure to modifiable environmental contributors [3].

### 7.1. Environmental Factors Contributing to the Occurrence of CHDs

Environmental factors contributing to the occurrence of CHDs are extremely heterogeneous and can be broadly classified into extrinsic and intrinsic factors.

#### 7.1.1. Extrinsic Factors

Extrinsic factors are generally considered highly modifiable, and there has been a consensus to identify and prevent such exposures in order to decrease the associated incidence of CHDs. Extrinsic environmental factors include prenatal maternal exposure to heavy metals (lead, cadmium, mercury, and arsenic), organic solvents (cleaning fluids), various therapeutic drugs (thalidomide, anticonvulsants, and antidepressants), nutritional deficiencies (folic acid/vitamin B12, B3, D, and iron), and retinol/vitamin A, as well as alcohol consumption and marijuana use [4,6,7]. Parental exposure to toxicants (such as paternal smoking) has been demonstrated to increase the risk of having children with CHDs [109].

A conscious lifestyle can modify the risk of exposure to these environmental factors and reduce the incidence of associated CHDs. The emerging body of recent empirical studies suggests that air pollution is an important extrinsic contributor to the development of CHDs, especially when this exposure occurs during the months of preconception and continues to around eight weeks’ gestation [110,111,112,113,114]. There are significant associations between increased risk of specific CHD subtypes and exposure to fine particulate matter with a diameter ≤ 5 μm and ≤10 μm, NO_2_, CO, O_3_, SO_2_, or polycyclic aromatic hydrocarbons [111,112,113,115,116]. High versus low CO and SO_2_ exposure was associated with an increased risk of ToF, whereas particulate matter and ozone (O_3_) increased the risk of ASDs and categorical NO2 exposure was associated with an increased risk of CoA [112,115]. Exposure to air pollutants may alter epigenetic modifications such as DNA methylation, which, in turn, may affect inflammation, leading to increased oxidative stress and mitochondrial dysfunction, and result in cardiac malformations [117,118]. It has been reported that gestational exposure to particulate matter ≤ 5 μm may increase the risk of mutations in the transcription factors GATA4 and NKX2.5, which are involved in fetal cardiac development and may cause CHDs in offspring [119,120,121,122]. Recent investigations by Yan et al. [123] have summarized five primary mechanisms through which fine particulate matter (PM_2.5_) affects adverse birth outcomes. These are: transcriptional and translational regulation, oxidative stress (OS) and inflammatory responses, and epigenetic regulation.

Other major pollutants in modern times are micro- and nano-plastics. A recent study using chick embryos showed that nano-plastics are capable of dysregulating neural crest cell migration, resulting in congenital cardiac and craniofacial anomalies [124].

#### 7.1.2. Intrinsic Factors

Intrinsic factors are much more difficult to control because they relate to internal physiologic conditions during embryogenesis, including maternal age, diseases and infections. These are generally regarded as less modifiable than the external environment and represent the complex interplay of biological processes that occur between the maternal intrauterine environment and the fetus in the course of fetal development and may alter the intrauterine environment of the mother [4].

Moreover, recent studies suggest that abnormal function and inflammation of the placenta is sufficient to lead to CHD, and there exists a placenta–heart axis that must be maintained for normal cardiac development [28,29]. In fact, impaired maternal metabolism via maternal microbiome profiling has been linked with several subtypes of CHDs indicative of an unfavorable maternal environment during cardiogenesis [125,126,127].

Due to the fact that increasing numbers of women in developed countries are delaying childbearing to an older age, maternal age has increased in the last decades, consequently causing a higher birth prevalence of CHDs [9,128,129]. Studies have shown that advanced maternal age (>35 years) is associated with an increased prevalence of several CHD phenotypes: laterality defects (aPR = 2.06; CI: 1.22–3.48), all conotruncal defects (aPR = 1.30; CI: 1.03–1.65), and specifically D-TGA (aPR = 1.65; CI: 1.10–2.48), CoA (aPR = 1.54; CI: 1.10–2.16), VSDs (aPR = 1.20; CI: 1.06–1.36), and ASDs (aPR = 1.36; CI: 1.05–1.77). The significant adjusted incidence rates ranged from 1.20 to 2.06 [130]. Similarly, Reefhuis and Honein [131] observed that advanced maternal age (35–40 years) was associated with all heart defects (OR = 1.12, 95% CI: 1.03–1.22).

Among environmental contributors, maternal PGDM is a highly prevalent and well-established risk factor for CHD, increasing 3- to 5-fold the risk of having an infant with CHD [4,22,23,24]. Meta-analyses carried out by several groups have identified the increased likelihood of specific CHD phenotypes resulting from exposure to maternal PGDM, including TGA, PTA, heterotaxy, VSDs and ASDs [37,38]. The incidence rates of CHD from pregestational type 1 and type 2 DM are similar, but type 1 has the highest risk for conotruncal defects (CTDs) and AVSDs, while type 2 is more associated with heterotaxy and LVOTO malformations [39]. However, the association between gestational diabetes mellitus (GDM) and CHD in offspring was inconsistent in the conducted meta-analyses. An analysis according to types of CHD showed that maternal GDM was associated with higher risks of ASDs, VSDs, and ToF [41]. However, inconsistent results were obtained in the studies evaluating the association between GDM and different CHD phenotypes in offspring. Chen et al. [42] suggested that the risk of CHD in newborns of mothers with PGDM was significantly higher than in newborns of mothers with GDM.

Similarly, maternal obesity, particularly obesity which comes along with other health complications, such as T2DM, has also been independently linked to CHDs [19,20]. The increased risk associated with maternal obesity includes a wide range of different heart defects, including TGA, ToF, septal defects, aortic arch defects, PDA, LVOTOs, RVOTOs and UVH [21,30,31].

Other maternal pathologies include infections such as rubella and cytomegalovirus, as well as phenylketonuria [4,6]. Recently, an increased incidence of CHDs was reported in cases of maternal COVID-19 infections [132].

The use of assisted reproductive techniques (ARTs) has also been proposed as one of the potential contributory causes of the development of CHDs in newborns, although the risk varies with the assisted reproductive technique applied [133,134,135]. The authors speculate that this may not only be due to the reproductive technology but also due to the underlying reason for the infertility of the couple being strongly related to the environmental conditions. This indicates the need for further research to validate the actual evidence and state the real risk of CHDs following ART pregnancies.

Table 3 presents selected common environmental (teratogenic) factors and the associated CHDs occurring in the fetus.

Although maternal factors are recognized risk factors for CHDs, the underlying molecular mechanisms remain unclear [19,26,27]. The list of environmental factors that can potentially cause CHD is nonexhaustive. The rapid transformation of society and its subsequent influence on our environment and lifestyle will continually give rise to other unknown CHD risk factors.

### 7.2. Familial Risk of Inheriting Congenital Heart Defects

The familial risk of CHDs in children is a serious concern for families when either parent has a CHD or when a sibling is born with a CHD. The presence of CHDs in parents significantly elevates the risk for their offspring, with a baby’s risk being increased 3-fold if either parent has been diagnosed with a CHD. Research has demonstrated that if a mother has a CHD, the risk of her child being born with a CHD ranges from 2.5% to 18%, with an average risk of 6.7% [136]. In contrast, if a father has a CHD, the risk for the child is lower, ranging from 1.5% to 3% [136]. Anomalies with the highest recurrence risk include heterotaxy, RVOTOs and LVOTOs [137].

Studies have shown that if one child has had a CHD, the chance of another child being born with the condition ranges from 1.5% to 5%, depending on the type of CHD in the first child [136]. This risk increases further if two or more children have CHDs, with an estimated recurrence rate of 5% to 10% for subsequent births [136].

A large cohort study reported a 60% greater risk of CHD among monochorionic and diamniotic twins [138]. Monochorionicity resulted in a 9-fold increased incidence of CHD. Abnormal placentation and, consequently, an abnormal blood flow distribution among the twins can result in twin–twin transfusion syndrome. A 13-fold increase in the risk of CHD is associated with this syndrome alone, but specific heart defects vary between twins [138]. In donor twins, there is an increased incidence of valvular stenosis and hypoplastic ventricles associated with reduced hemodynamic flow. Among recipient twins, the opposite effect is observed, where the prevalence of obstructive lesions and valvular regurgitation is increased [139]. Since both individuals are assumed to be genetically identical, CHD malformations are likely to result directly from epigenetic modifications, environmental factors, or abnormal placentation.

Some heart defects, such as those with autosomal-dominant inheritance, can arouse particular concern, as a parent with the defect has a 50% chance of passing the defect to each child, regardless of whether it is a male or a female [140]. The above means that there is also a 50% chance that the offspring will not be affected by the defect [140]. Notably, autosomal-dominant inheritance affects males and females equally, making this pattern of inheritance particularly relevant for families with CHDs.

The elevated recurrence rate of CHD in affected families indicates the need to identify its underlying genetic factors. Besides chromosomal abnormalities and copy number variation, several genetic variants linked to CHD were historically believed to follow a monogenic disease inheritance pattern. However, recent technological advances have allowed the identification of novel CHD variants and non-Mendelian genetic mechanisms underlying CHD.

### 7.3. Genetic Causes of Congenital Heart Defects (CHDs)

**Figure 6:**
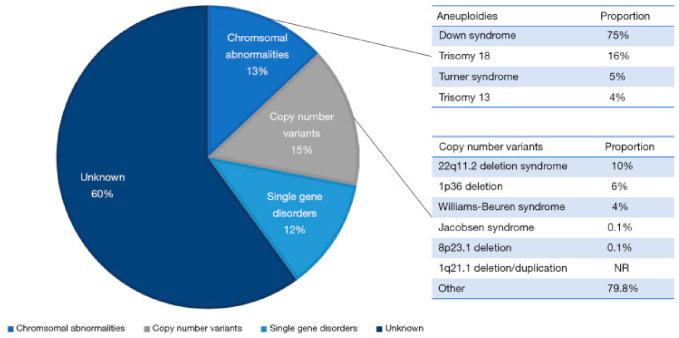
Specific genetic causes of congenital heart disease. Chromosomal abnormalities, copy number variations and single-gene variants are associated with ~40% of congenital heart disease cases, but the genetic causes of the majority (60%) of congenital heart disease cases remain unknown. All percentages are approximates based on recent publications. NR, not reported. This figure has been taken from the article of Yasuhara and Garg [10] distributed under the terms and conditions of the Creative Commons Attribution License (CC BY-NC-ND/4.0).

Genetic causes are considered to be the main factor in the pathogenesis of CHDs, but the genetic penetrance of variants to the phenotype and clinical course vary significantly [12]. Specific genetic causes can be detected in an estimated ~40% of CHD cases (Figure 6). These include chromosomal anomalies or aneuploidies accounting for ~13% of CHD cases (range: 9% to 18%) and copy number variants (CNVs) estimated at 10–15% (range: 3% to 25%) in syndromic CHD and 3% to 10% in non-syndromic CHD, as well as single-gene disorders (12%) (Figure 6) [10,11,141,142].

The genetic basis of CHD can be divided into syndromic CHD and non-syndromic CHD, where congenital abnormalities are isolated to the heart. In non-syndromic and isolated (without a familial history or a Mendelian inheritance) forms of CHDs, a multifactorial pathogenesis with interplay between inherited and non-inherited causes is recognized. Numerous genes have been discovered to be implicated in the pathogenesis of syndromic CHD. However, identification of the genetic contributors of non-syndromic CHD is more challenging because of genetic heterogeneity, incomplete segregation, and potentially oligogenic or polygenic origins [10,11]. A single candidate gene or genetic variant can produce a spectrum of heart malformations. Moreover, it may even occur in phenotypically normal humans [5].

#### 7.3.1. Genetic Abnormalities Associated with Syndromic CHD

Chromosomal abnormalities and copy number variation (CNV) have been strongly associated with syndromic CHD, due to disrupted expression and dosage of crucial developmental genes located within the affected loci [5].

Chromosomal mutations include either anomalies involving the loss or gain of complete chromosomes (aneuploidy), or structural abnormalities in subchromosomal regions (e.g., CNVs). Chromosomal aneuploidies include trisomies (13, 18 and 21) and monosomies such as Turner syndrome. Chromosomal abnormalities are a significant risk factor for CHDs, particularly in individuals with Down syndrome. The extra 21st chromosome present in individuals with Down syndrome is thought to contribute to the development of a diverse range of heart conditions observed in children with this condition [10,143]. Chromosomal aneuploidies and larger CNVs can be identified using karyotyping and chromosomal microarray.

CNVs are large deletions or duplications of DNA segments, and pathogenic CNVs are associated with syndromic CHDs. These include 22q11.2 deletion syndrome (DiGeorge syndrome), 1p36 deletion syndrome, 7q11.23 deletion (Williams–Beuren syndrome), terminal deletions of 11q (Jacobsen syndrome), 1q21.1 deletion/duplication, and 8p23.1 deletion syndrome, which can be detected by fluorescent in situ hybridization and/or chromosomal microarray (CMA) [10].

Single-gene defects play a significant role in the development of CHDs, accounting for approximately 12% of cases [10]. These defects often follow a Mendelian inheritance pattern, most commonly exhibiting an autosomal-dominant trait [144], which implies that only one copy of the mutated gene is necessary to manifest the condition. There are several examples of Mendelian syndromes associated with CHDs, including Alagille syndrome, Holt–Oram syndrome, Costello syndrome, Kabuki syndrome, Char syndrome and Noonan syndrome [10,144]. Table 4 shows the most common syndromic conditions associated with CHDs.

Recent advances in cytogenetics and molecular diagnostic techniques have significantly contributed to the identification of new genes and chromosomal regions involved in both syndromic and non-syndromic CHD. The knowledge of them enables a more defined explanation of the underlying pathogenetic mechanisms of CHD [3,10].

#### 7.3.2. Genetic Abnormalities Associated with Non-Syndromic CHD

In recent years, remarkable advances in next-generation sequencing (NGS) technologies have allowed for the identification of novel genetic etiologies for CHD and better understanding of the complex genetic architecture of non-syndromic CHD. Pathogenic variants that result in non-syndromic CHD can be broadly divided into 1) cardiac transcription factors, governing cardiac development (NKX2.5, GATA4, and members of the T-box family [TBX1 and TBX5]; (2) cardiac structural proteins (MYH6, ACTC1, and ELN); and (3) cell signal factors (neurogenic locus notch homolog protein 1 and vascular endothelial growth factor) [5,12,83,145,146,147,148].

Although mutations in the coding sequence or gene regulatory sequences affecting their expression can result in cardiac malformations, the precise genotype/phenotype relationship is often challenging to establish [149,150]. Genetic variants are implicated as the primary drivers of pathogenesis, but genetic penetrance of variants to phenotype and clinical course varies significantly. Table 5 shows selected monogenic causes of non-syndromic CHDs.

Notably, even though more than 400 genes associated with CHDs have been identified, only about one-third of cases have a simple genetic cause, highlighting the substantial contribution of multifactorial inheritance to the disease [3,4,5]. Additionally, mutations in specific genes such as the Notch receptor (*NOTH*) family, the NKX2 homeobox (*NKX2.5*) family, the GATA-binding protein 4 (*GATA4*) family and the T-box transcription factor (*TBX*) family have been identified as contributing factors to non-syndromic CHDs [9,144,145,151,152].

*NKX2.5* is the master regulator of cardiac development. Mutations in *NKX2.5* are often associated in humans with ASDs and conduction abnormalities. *NKX2.5* gene mutations have also been reported in VSDs, ToF, aortic stenosis, and HLHS, demonstrating that *NKX2.5* has multiple roles during heart development [10,12,153].

*GATA4* is a potent activator of many cardiac genes, including the genes encoding natriuretic peptides (NPPA and NPPB), cardiac myosin heavy chains (MYH6 and MYH7), and troponin isoforms (TNNI3 and TNNC1), as well as the cardiac muscarinic m2 acetylcholine receptor (CHRM2). *GATA4* is a mutual cofactor of *NKX2.5* [12,154]. Over 100 *GATA4* mutations linked to various cardiac defects and types of CHD have been identified to date. *GATA4* is necessary for cardiac septation in a subset of cardiac progenitor cells called the “second heart field” [155]. Variants of *GATA4* have been associated with ASDs, VSDs, TOF, PDA and pulmonary stenosis (PS) [10,12,156,157].

In human hearts, TBX5 is expressed in the epicardium, myocardium, and endocardium of both embryonic and adult hearts [158,159]. The T-box family of transcription factors plays an important role in patterning the embryonic germ layers. It functions as a transcriptional activator, interacting with various cofactors, including *GATA4*, *NKX2.5*, *MEF2C* and *SALL4*, to regulate genes essential for cardiac morphogenesis and development of the conduction system [160]. Variants in TBX1 in humans are commonly associated with abnormalities in pharyngeal arch patterning and ventricular septation [10,12].

Despite tremendous advances in genetic sequencing technologies, the identification of pathogenic variants in patients with non-syndromic CHD has been challenging, even in familial cases of CHD. Direct analyses of human CHD tissues, comprehensive assessments of non-coding sequences and oligogenic variants, and examinations of the effects of environmental exposures may be useful in diagnostics, classification, and clinical care of patients with CHD, but also for better understanding of developmental and genomic biology.

### 7.4. Epigenetic Modifications in CHDs

Epigenetic modification is an extragenomic mechanism that does not affect a DNA sequence but is capable of regulating gene expression by influencing transcription or inhibiting translation [13,78,161]. There is growing evidence demonstrating the association of epigenetics with the development of polygenic diseases, including CHDs. To understand the mechanisms whereby gene–environment interactions lead to complex CHDs, epigenetic mechanisms such as DNA methylation, histone modifications, chromatin remodeling and non-coding RNAs must be considered [12,13,162].

Methylation of DNA is the best-characterized epigenetic mechanism. Tightly controlled DNA methylation is essential in early fetal development and in the regulation of genomic programing. DNA methylation patterns are tissue-specific and vary depending on cell type. [162,163]. In 2011, Chowdhury et al. [164] provided the first evidence of association between maternal gene-specific DNA methylation and CHD through a case–control study of genome-wide maternal DNA methylation. Other authors reported methylation levels of APOA5 and PCSK9 to be elevated in neonates with aortic valve stenosis (AS), potentially indicating risk factors for adult CHD [165,166]. Patients with ToF and congenital VSDs demonstrated hypermethylation in the promoter region of cytochrome C oxidative synthase protein (SCO2) [167]. Maternal hypermethylation of LINE-1 DNA has also been found to be related to a heightened risk of non-syndromic coronary heart disease. The MTHFR C677T mutation has been demonstrated to be associated with up to 50% of certain CHDs [168]. Among methylated genes, a strong association between prenatal placental DNA methylation and congenital heart disease (CHD) has been revealed by Gene Ontology (GO) analysis, with certain genes such as *TLL1*, *CRABP1*, *FDFT1* and *PCK2* located within the differentially methylated regions associated with clinical phenotypes [167].

Histone modifications are crucial for regulating gene expression by altering chromatin structure, which affects the accessibility of DNA for transcription. In patients with CHD, a marked excess of de novo variants affecting histone-modifying genes, leading to altered methylation, particularly of genes heavily expressed in the heart, has been demonstrated [166,169]. Significant de novo mutations in genes responsible for the writing, erasing, and reading of H3K4 methylation during H3K4 methylation or H2BK120 ubiquitination have been identified. Decreased acetylation of H3K4, H3K9, and H3K27 after the downregulation of histone acetyltransferase has been linked to CHD through decreased *GATA4* expression [170]. A lack of histone deacetylase 2 has been shown to cause severe cardiac developmental defects and myocyte hyperproliferation, possibly through hyperacetylation of *GATA4* [171]. These findings suggest a potential pathogenic role of abnormal histone methylation in coronary heart disease [169].

Defects in DNA remodeling complexes have also been associated with CHD [172,173,174]. Chromatin remodeling refers to the process of repositioning, ejecting, and restructuring nucleosomes, thus regulating the accessibility of DNA sequences to the transcription machinery [175]. Several studies in mice have shown that a deficiency in Brg1, which encodes an ATPase subunit of a DNA remodeling complex essential for cardiac development, causes congenital heart defects [172,176]. A recent case–control study found significantly lower levels of *BRG1* expression in the myocardium of patients with CHDs compared with controls [173]. Interestingly, *GATA4* expression was directly correlated with *BRG1* expression levels in the myocardium of patients with CHDs, suggesting that the pathogenic effects of *BRG1* deficiency could be due to its impact on *GATA4* expression. Mutations in another chromatin remodeler, *CHD7*, have also been associated with CHD, specifically AVSDs and CTDs [177].

Non-coding RNAs (ncRNAs), including microRNAs (miRNAs) and long ncRNAs (lncRNAs), are other epigenetic regulators crucial for normal cardiac development. miRNAs inhibit translation by binding to target mRNAs, whereas lncRNAs can directly engage with chromatin remodeling complexes to regulate transcription [178,179,180,181].

Several studies have identified irregular miRNA expression in ToF patients, with the implication of miR-421 in *SOX4* regulation [182]. The overexpression of miR-424 was associated with increased proliferation and decreased expression of *HAS2* and *NF1* in right ventricular cardiomyocytes from ToF patients [17,18,183].

Both high and low miRNA expression levels have been demonstrated to cause CHDs [15,16,17,18]. For example, excess miR-1 miRNA abundantly expressed in the heart suppressed ventricular cardiomyocyte proliferation, while targeted miR-1-2 deletion caused VSDs [15,184,185]. The above results can be attributed to altered expression levels of miRNA targets, such as the transcription factor Hand2 and the histone-modifying protein HDAC4 [15,184,185]. The repression of histone deacetylase4 (HDAC4) enhances the activity of the myocyte enhancer factor 2A (MEF2) transcription factor, which, in turn, facilitates the expression of miR-1 [184,185].

The downregulation of miR-206 and miR-240 and upregulation of miR-424/424 and miR-421 have also been associated with ToF by causing changes in the expression of the target genes involved in intercellular communication (*GJA1*), cardiac septation (*NF1* and *HAS2*), and the development of the cardiac outflow tract (*JARDI2*) [16,17,18,186]. The most common miRNAs associated with CHDs are presented in Table 6.

During the early stages of cardiac development, lncRNAs regulate the differentiation of pluripotent stem cells and cardiac precursors into mature cardiac cells [188]. Then, lncRNAs oversee cellular senescence and other pathways that are pivotal in cardiac pathology [189]. Hundreds of lncRNAs have been reported to date to be associated with cardiac development and diseases [178,179,180,181]. The low endogenous expression level of these lncRNAs and the complexity of identifying their binding partners makes the elucidation of their functional roles a significant challenge [190].

Recent advances have focused on utilizing epigenetic biomarkers for the diagnosis of CHD. As potential diagnostic indicators, aberrant DNA methylation patterns and circulating miRNAs are being investigated [191]. In ToF cases, a genome-wide methylation assay identified 25 genes with high predictive accuracy for ToF [192], while in ventricular septal defect cases it revealed 80 CpG sites in 80 genes highly accurate in predicting VSDs [193]. Circulating miRNAs, such as hsa-let-7a and hsa-let-7b, show a diagnostic value for atrial septal defects [191]. Researchers have found a panel of four miRNAs (miR-19b, miR-22, miR-29c, and miR-375), which were remarkably upregulated in pregnant women who had fetuses with CHDs at 18 to 22 weeks of gestation. Moreover, the combination of the four miRNAs exhibited high efficiency for the early diagnosis of fetal CHD [194,195].

Beyond enhancing our understanding of disease processes, epigenetics may play a pivotal role in advancing innovative treatments and diagnostic approaches.

## 8. Genomic Techniques

In CHD, clinically overt cases with a high index of suspicion for single-gene defects are increasingly subject to conventional genetic diagnosis using such techniques as karyotyping, which facilitates the visualization and analysis of chromosomal composition at the macroscopic level to elucidate chromosomal aberrations, including aneuploidies and fluorescence in situ hybridization (FISH), enabling the visualization and subsequent detection of the presence and location of specific DNA sequences on chromosomes and in cell nuclei associated with CHD.

Chromosomal microarray analysis (CMA) is a high-resolution genome-wide cytogenetic technique used for the detection of submicroscopic chromosomal imbalances, including copy number variants (CNVs), across the entire genome. Clinical genetic testing in infants with CHDs using karyotyping, FISH, and CMA has an overall diagnostic yield of 15–25%, with a higher likelihood of finding a genetic diagnosis in patients with dysmorphic facial features and extracardiac anomalies [196,197].

Genomic technology has transformed our understanding of CHDs and associated gene–environment interaction. Techniques such as genome-wide association studies (GWASs), single-nucleotide variations (SNVs), and copy number variants (CNVs) have been instrumental in identifying genetic risk factors for CHDs [198,199,200]. The state-of-the-art techniques, such as next-generation sequencing (NGS), single-cell RNA sequencing (scRNA-seq), microRNA sequencing (miRNA-seq), human-induced pluripotent stem cells (hiPSCs), cytogenetics, and epigenetics, have facilitated the discovery of the role of individual cells during cardiac development and provided novel insights into the genetic architecture of CHDs [11,12,201].

Advancements will improve whole-genome sequencing/whole-exome sequencing (WGS/WES), revealing de novo variants (DNVs) and supporting data processing, leading to the development of increasingly accurate prognostic prediction tools.

Next-generation sequencing (NGS) technologies are unravelling the role of oligogenic inheritance, epigenetic modification, genetic mosaicism, and non-coding variants in controlling the expression of candidate CHD-associated genes [11,77,148]. Owing to these molecular diagnostic techniques, new genes and chromosomal regions involved in syndromic and non-syndromic CHD have been identified. Advances in sequencing technologies and functional genomic models of CHD will allow for the integration of genome editing, cardiac bioengineering and cardiac organoid models. The development of these technologies will enable the elaboration of new regenerative and preventive therapeutic approaches to treat the core disease mechanisms in CHD patients in the future. However, the prediction of clinical risk based on these factors remains challenging because the underlying cause of more than half of CHD cases remains unknown [10]. The indications for genetic testing and associated CHD conditions are summarized in Table 7.

Examples of metabolic disorders are diabetes, obesity, dyslipidemia, metabolic syndrome, nonalcoholic fatty liver disease, and insulin resistance.

## 9. Diabetes Mellitus (DM)

### 9.1. Definition and Classification of Diabetes

Diabetes mellitus (DM) is a metabolic disorder characterized by hyperglycemia resulting from an insulin secretion defect, insulin action disorder, or both [202,203,204].

DM can be classified into the following general categories: (1) Type 1 diabetes mellitus (T1DM) (insulin-dependent) is associated with autoimmunity against pancreatic beta cells, i.e., the destruction of beta cells caused by the expression of autoantibodies against insulin (IAAs), antibodies against insular cells (ICAs), antibodies associated with insulinoma protein-2 antibodies (IA-2As), glutamic acid decarboxylase antibodies (GADAs) and zinc transporter antibodies 8 (ZnT8As); insufficient insulin secretion; and hyperglycemia [205,206]. T1DM is especially prevalent among young people and accounts for approximately 5% of all types of diabetes. (2) Type 2 diabetes mellitus (T2DM) (insulin-independent) is characterized by insulin resistance, with the body being unable to use secreted insulin such that symptoms of hyperglycemia occur. T2DM is more common in older and overweight people and affects most diabetic individuals worldwide (90–95%) [207]. It is also noteworthy that in recent years, in younger people (<40 years of age), a two- or three-fold increase in the incidence of T2DM has been noted [208]. (3) Gestational diabetes mellitus (GDM) is defined as a glucose metabolism disorder of both insulin resistance and β-cell dysfunction, with the onset or first recognition occurring during pregnancy.

Reduced insulin-stimulated glucose uptake further contributes to hyperglycemia, overburdening the β-cells, which have to produce additional insulin in response [209,210]. According to the American Diabetes Association (ADA) “Standards of Medical Care in Diabetes” of 2022, GDM is defined as diabetes diagnosed in the second trimester (13–27 gestational weeks) or the third trimester (≥28 gestational weeks) that was not clearly overt diabetes prior to gestation [211]. More and more women are at risk of developing GDM due to the obesity epidemic and late motherhood. It occurs transiently in the 3–9% of pregnant women affected and usually disappears completely after delivery [212]. (4) Secondary types of diabetes, which include several specific causes, such as impaired beta cell function due to monogenic defects, pancreatic diseases and drug/chemical-induced endocrinopathies, appear in 2–5% of individuals [211,213,214,215].

The term matDM encompasses the three clinical types of diabetes, T1DM, T2DM and GDM, encountered during pregnancy [216].

For women with pre-existing diabetes (both type 1 and type 2) who are planning a pregnancy, the classification developed by Priscilla White [217] is currently used. This classification takes into account the age of onset, the duration of diabetes, the presence of any vascular complications, and the need for insulin treatment. Based on this, the patient is assigned to one of nine categories. The classification of gestational diabetes proposed by White is presented in Table 8.

The aforementioned classes, except for A, require insulin therapy to obtain normoglycemia. This classification system is widely used to assess the risk to the mother and fetus based on the maternal risk factors. The White classification allows assessment of the course of pregnancy both in women who are just planning to have children and in those who are already expecting a child.

Hyperglycemia first diagnosed during pregnancy should be diagnosed and classified according criteria for diagnosis of gestational diabetes presented in Table 9. The GDM diagnostic criteria, according to the recommendations of the WHO and the Polish Diabetes Association (PDA—in accordance with PDA guidelines 2024/2025), are unified and based on performing an oral glucose load test (OGTT) between 24 and 28 weeks of gestation (or earlier if there is a risk). 

### 9.2. The Incidence of Diabetes (DM)

The incidence of DM has increased dramatically in recent decades, making the condition a significant public health concern. Current estimates suggest that the global prevalence of diabetes has reached 6.1% [219]. It is estimated that 537 million people worldwide (aged between 20 and 79), or approximately 1 out of 10 adults, have diabetes, and it is predicted that this will rise to 643 million by 2030 and 783 million by 2045, making it one of the fastest growing diseases worldwide [204,220]. Accordingly, 129.4 million women of childbearing age (20–49 years) are affected by DM, and 20.9 million live births worldwide are at risk of maternal hyperglycemia [24].

The global incidence of symptomatic T1DM is markedly varied. It was estimated that in 2025 there were 9.5 million people living with T1DM globally (compared to 8.4 million in 2021, a 13% increase), with 1.0 million of those aged 0–14 and 0.8 million aged 15–19 years. In lower-income countries, the prevalence has increased by 20% from 1.8 million in 2021 to 2.1 million in 2025. Incident cases in 2025 are estimated at 513,000 (164,000 aged 0–14 and 58,000 aged 15–19 years), with the incidence having increased by 2.4% in the last year. The projected T1DM population for 2040 is estimated to be 14.7 million [221].

The most common type of diabetes mellitus is T2DM. In 2017, approximately 6.28% of the world’s population was affected by T2DM, and around 1 million deaths yearly can be attributed to T2DM alone [222].

On a global scale, in 2021, an estimated 21.1 million or approximately 16.7% of births were adversely affected by diabetes in pregnancy. Of these, 80.3% were classified as GDM; 10.6% as diabetes detected before pregnancy, including type 1 or type 2 diabetes mellitus; and the remaining 9.1% were cases of diabetes first detected during pregnancy (including type 1 and type 2 diabetes) [223,224].

**Figure 7:**
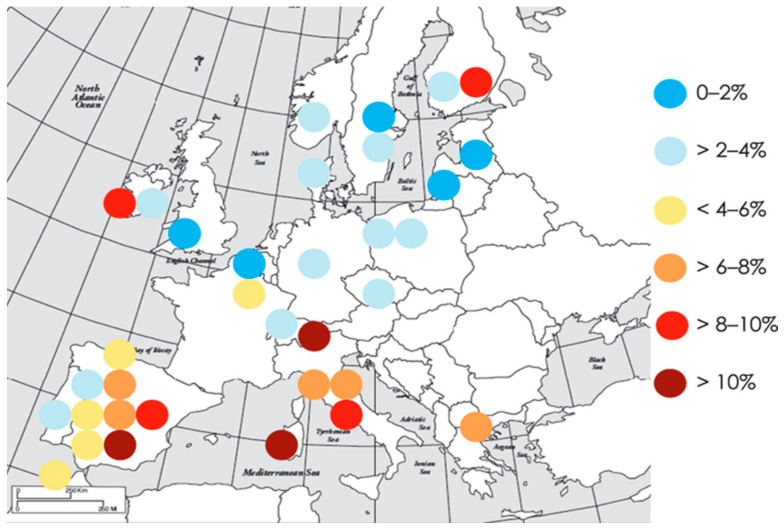
Reported prevalence of gestational diabetes in Europe. This figure has been taken from the article of Buckley et al. [225] distributed under the terms and conditions of the Creative Commons Attribution License (CC BY-NC-ND/4.0).

Gestational diabetes mellitus (GDM) is diagnosed in 5.8–12.9% of pregnant women. Pregestational diabetes mellitus (PGDM) occurs in 0.4–1.1% of pregnant women [202,203,204]. The prevalence of GDM varies substantially between populations, with a range of 1.7–11.6% (Figure 7). The prevalence seems to vary considerably within Europe, with a trend towards lower prevalence of GDM in Northern or Atlantic seaboard parts of Europe, where the estimates are mostly lower than 4%, whereas in the South or Mediterranean seaboard region, estimates higher than 6% predominate (Figure 7).

The reported incidence of GDM varies widely by world region, race/ethnicity and the socioeconomic status of patients.

In Europe, it occurs in 3–5.4% of pregnant women [226]. GDM in Europe has been found to be more common among Asian women than among European women [227]. In Poland, the incidence of GDM is 6.2%, while for PGDM it is 1% [228].

In the United States (US), diabetes affects more than 25 million Americans, or over 8% of the current population, and this figure is predicted to reach 10.8% in 2050 [229,230]. Even within countries, the incidence of GDM varies. In the US, Native American, Asian, Hispanic, and African-American women are at higher risk for GDM than non-Hispanic Caucasian women [231,232,233,234,235,236,237,238,239]. In Australia, the GDM prevalence was found to be higher in women whose country of birth was China or India than in women born in Europe or Northern Africa [240]. It was also higher in Aboriginal women than in non-Aboriginal women [202,241]. The frequency of GDM worldwide is highest in Southeast Asia (26.6%). The proportion of pregnancies complicated by GDM in Asian countries has been reported to be lower than the proportion observed in Asian women living in other continents [242]. GDM is least common in the Chinese population of Taiwan and southern India. In India, GDM has been found to be more common in women living in urban areas than in women living in rural areas [243]. The prevalence of diabetes mellitus among women aged 20–39 years is low in Japan; the prevalence of strongly suspected diabetes was 1.9% according to the National Health and Nutrition Survey Japan in 2019 [244,245]. The estimated prevalence of GDM in Japanese pregnant women based on the criteria issued by the Japan Diabetes Society was 2.4–6.6% [246].

Øyen et al. [23] describe the results of a large national cohort study in Denmark that examined trends in the prevalence of CHD cases attributable to PGDM among 2,025,727 births from 1998 to 2011, with a low prevalence of exposure to maternal PGDM (0.36%) in comparison with that reported in North America (>1%). The prevalence of births with PGDM in this cohort did demonstrate an increase over time (from 0.23% in 1978–1986 to 0.42% in 1994–2011), and, in comparison with the overall prevalence of CHD among births to mothers without pregestational diabetes (8/1000), the overall prevalence of CHD among births to mothers with PGDM was four times higher, which is consistent with previous observations. A finding of this study that causes concern was that the high proportion of CHDs attributable to PGDM among births to mothers with PGDM in 1978 to 1986 (79%) showed only a 6% decline by the end of the study period from 1997 to 2011 (to 74%). The authors were able to corroborate the strong associations between maternal PGDM and CHDs reported in previous case–control studies, that the association of PGDM with CHDs is evident for specific CHD phenotypes and groupings of CHD phenotypes, that this association is stronger for certain CHD phenotype groupings (e.g., heterotaxia and conotruncal defects) than for others, and that diabetes mellitus is also associated with noncardiac defects.

There is also some evidence that GDM prevalence varies by season, with more diagnoses of GDM in summer than winter [247]. The discrepancies in the data may be caused primarily by different diagnostic methods for diagnosing carbohydrate metabolism disorders, different diagnostic criteria, as well as cultural and ethnic differences in the studied populations.

### 9.3. The Role of Glucose Metabolism in a Normal Pregnancy

In the course of pregnancy, the mother’s body undergoes a series of physiological changes in order to support the demands of the growing fetus. A growing fetus requires constant access to nutrients. During early embryogenesis, glucose is the main source of energy [248,249]. In the late-embryonic, fetal and neonatal stages, there is a switch in the energy substrate of cardiomyocytes to fatty acid metabolism as the main source of cellular energy (ATP) [250,251]. This metabolic shift is due to adverse maternal environments and changes in the expression of genes encoding metabolic enzymes and transporters, and it plays a key role in fetal heart maturation [252,253].

Several glucose transporter proteins are responsible for the facilitated diffusion of glucose from the maternal to the fetal circulation via the placental barrier. Glucose transporter type 1 (GLUT1), insulin-independent, ensures a constant supply of glucose to cells, irrespective of insulin levels. It is encoded by the Solute Carrier Family 1 Member 2 (Slc1a2) gene and is considered the major transporter isoform in early embryogenesis [254,255,256]. Throughout development, GLUT1 is gradually downregulated with concomitant upregulation of glucose transporter type 4 (GLUT4), an insulin-dependent glucose transporter which translocates from intracellular compartments upon insulin binding to the insulin receptor in muscle, which allows for glucose uptake. GLUT 4 becomes predominant by the end of gestation [257,258]. Over the course of gestation, insulin sensitivity shifts depending on the requirements of pregnancy. With gestational age, insulin resistance (IR) increases. As a result of this process, a growing fetus has constant access to energy [259]. This forces the flow of energetic substrates through the placenta [255]. The level of glucose in the blood of the fetus is adequate to the level of glucose in the mother, but slightly lower. Despite increased gluconeogenesis, glycemic values are lower in healthy pregnant women. This is due to an increase in insulin secretion and increased glucose consumption by fetal tissues [256]. The common denominator of diabetes is chronic hyperglycemia resulting from an insufficient amount of insulin. In the case of the IR-related substrate, hyperglycemia is a consequence of its relative deficiency. During pregnancy, physiological insulin resistance increases. This is related to ensuring greater availability of glucose for the fetus. This is due to the increased caloric needs of the pregnant woman; higher body weight; and the activity of placental hormones such as human placental lactogen, leptin, adiponectin, cortisol, progesterone, estrogens, placental growth hormone and prolactin [260]. The metabolic status of a pregnant woman leads to an increased amount of cholesterol, triglycerides and ketones, and these, passing easily through the placenta, stimulate the growth of the fetus [256,261]. In addition, during pregnancy, insulin degradation by the liver is reduced. To compensate for this insulin resistance, the pancreatic cells increase insulin secretion, thereby preventing the blood glucose concentration from exceeding normal values [260]. In the case of their failure, the regulation of carbohydrate metabolism is disturbed. As far as β cell dysfunction is concerned, it has been observed that it is reduced by 30–70% in GDM, which indicates that β cells are unable to compensate for the increase in insulin resistance, leading to the development of GDM. The mechanisms underlying β cell dysfunction are not fully understood, but they probably partially overlap with those described in T2DM diabetes [262,263,264].

These data suggest that high glucose suppresses cardiac maturation, providing a possible mechanistic basis for congenital heart defects in diabetic pregnancy.

### 9.4. Risk Factors for Diabetes Development

The risk factors for T2DM include complex combinations of genetic, metabolic and environmental factors that interact with one another and comprise both non-modifiable (ethnicity and family history/genetic predispositions) and modifiable risk factors (obesity, low physical activity, and an unhealthy diet high in sugars and fats, as well as smoking and excessive alcohol consumption). Risk factors for developing GDM include overweight and obesity; excessive gestational weight gain; a Westernized diet; ethnicity; genetic polymorphisms; advanced maternal age; intrauterine environment (low or high birthweight); family and personal history of GDM; and other diseases of insulin resistance, such as polycystic ovarian syndrome. These conditions affect cell function, resulting in a complex network of pathological changes that influence one another and lead to the perpetuation of insulin dysfunction [265]. GDM increases the risk of a number of short-term and long-term health consequences for both the mother and the fetus [256].

Recently, it has been suggested that maternal exposure to fine particulate matter (PM_2.5_) during the first trimester of pregnancy contributes to the development of GDM and is a risk factor for T2DM because it is associated with impaired glucose metabolism [266,267]. After being inhaled into the lungs, PM_2.5_ can enter the fetus through the air–blood barrier and placental barrier, generating large amounts of harmful products such as pro-inflammatory cytokines and reactive oxygen species, thereby causing cardiac developmental toxicity to the fetus. The underlying mechanisms involved include: interference with genes related to cardiac development, dysfunction of genes associated with heart function, OS, inflammation, mitochondrial impairment, epigenetic modification, endoplasmic reticulum stress, autophagy, apoptosis, Aryl hydrogen receptor signaling, Wnt signaling, DNA damage and disorders of Ca^2+^ homeostasis. It has been suggested that gestational exposure to PM_2.5_ increases the incidence of congenital diseases in offspring, including CHD. In addition, animal model studies have revealed that gestational exposure to PM_2.5_ can disrupt normal heart development in offspring, although the potential molecular mechanisms have yet to be fully elucidated [121].

Recent advances in microbiome research have provided new insights into the complex role of the gut microbiome in metabolic regulation [268,269,270,271,272]. Numerous studies have shown that the composition and functions of the gut microbiota may be important factors influencing the risk of development of diabetes. They indicate that changes in dysbiosis can promote IR and T2DM [265,273]. A high-fat diet can induce up to threefold production of lipopolysaccharides (from Gram-negative bacteria) in mouse models, thus contributing to low-grade inflammation and insulin resistance [274,275]. In addition, intestinal dysbiosis can reduce short-chain fatty acid synthesis that promotes gut barrier integrity, pancreatic β cell proliferation and insulin biosynthesis [276,277]. Dysbiosis can also compromise the production of other metabolites, such as branched amino acids and trimethylamine, disrupting glucose homeostasis and triggering T2DM development [278,279]. As a result, the gut microbiome is increasingly considered a potential target for new therapeutic strategies in the prevention and management of diabetes [269,280].

### 9.5. Mechanisms Influencing the Risk of CHD in the Offspring of Diabetic Mothers

The mechanism by which maternal diabetes increases CHD risk is far from clear and still a matter of debate [79]. This is because it is a complex metabolic disease. In addition to hyperglycemia, patients may also present with hyperlipidemia [281], protein misfolding and glycation [282,283], as well as impaired glucose tolerance and impaired insulin sensitivity [283]. Abnormal early pregnancy maternal lipid profiles have been associated with increased risk for CHD in offspring. Despite this assumption, there is no consensus on how hyperglycemia causes CHD. Extensive studies in a variety of animal models have led to many divergent hypotheses, including hypoxia and/or increased oxidative stress [284], activation of the polyol or hexosamine pathways [285,286], increased apoptosis [287], and endoplasmic reticulum stress [288,289]. In experimental studies using mice, oxidative stress and/or inflammation appeared to increase vascular insulin resistance (IR) and/or impair hepatic glucose metabolism [290,291].

**Figure 8:**
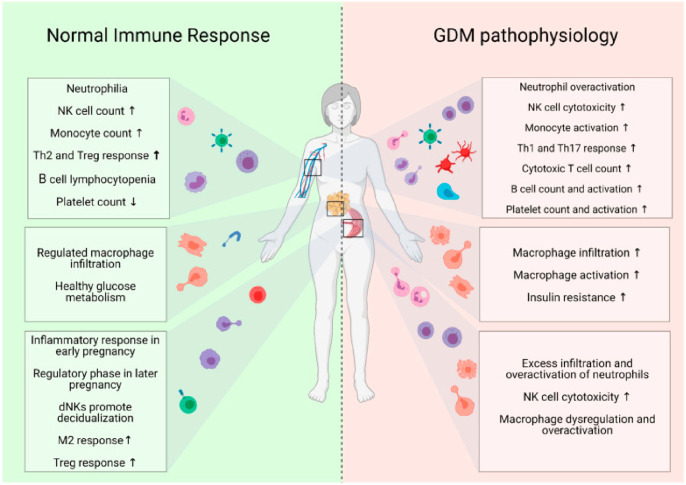
An overview of immune cell phenotypes in maternal circulation, adipose tissue and placental tissue in healthy uncomplicated pregnancy compared to pregnancy complicated by GDM. ↑, increased; ↓, decreased; GDM: gestational diabetes mellitus; NK: natural killer; Th2: T-helper 2 cell; Th1: T-helper 1 cell; Th17: T-helper 17 cell; Treg: regulatory T cell; dNK: decidual NK cell. This figure has been taken from the article of McElwain et al. [292] distributed under the terms and conditions of the Creative Commons Attribution License (CC BY-NC-ND/4.0/).

In GDM, low-grade inflammation arises and leads to the sequential activation of a variety of inflammatory mechanisms in maternal and gestational tissues [292,293]. Key maternal immunological mediators such as neutrophiles, monocytes/macrophages, natural killer (NK) cells and T cells must be balanced by the maternal immune system to avoid adverse pathology and/or complications of pregnancy [292]. The placenta is known to play an important role in shielding the fetus from the maternal immune system [294,295]. The accumulation of pro-inflammatory molecules in the placenta may be a cause of disruption of trophoblast physiology and a lower fetal-to-placenta weight ratio, an abnormal placenta villi structure and placenta vascularization, decreased apoptosis, increased autophagy and oxidative stress, as well as mitochondrial damage [292,296,297]. In an experiment using a human trophoblast cell line from the first trimester, hyperglycemia triggered trophoblast secretion of inflammatory cytokines, suggesting that excess glucose leads to trophoblast dysfunction and inhibits adequate placentation development [298]. The placenta requires an initial inflammatory response for tissue remodeling and the support of angiogenesis. Inflammation further worsens pancreatic β cell function and increases IR in peripheral tissues such as the muscles, adipose tissue and placenta [293,299,300]. The influence of circulating and tissue-infiltrating (i.e., visceral adipose tissue and placental tissue) mediators on immune system cell populations in normal pregnancies and those complicated by GDM is presented in Figure 8.

However, there is still uncertainty concerning specific immune cell populations in GDM pathology, such as how circulating levels of NKT cells and dendritic cells are influenced, the extent of macrophage infiltration and polarization in placental tissue, and the identity and role of additional immune populations in mediating adipose tissue inflammation and metabolic dysfunction [292].

Abnormal levels of various inflammatory mediators such as tumor necrosis factor-α (TNF-α), transforming growth factor-β (TGF-β), interleukins (ILs), and adhesion molecules cause an intracellular inflammatory response and block insulin signal transmission [301,302]. In high-glucose environments, levels of IL-6 and IL-1β are significantly increased, which is closely related to IR [303,304]. IL-1β not only inhibits insulin signal transduction in macrophages, causing abnormal insulin secretion [304], but also causes proinflammatory cells to migrate to islets and damage them [305]. Epidemiological studies have also demonstrated that a systemic inflammatory marker, C-reactive protein, is associated with an increase in glucose intolerance and IR [306,307].

In addition to inflammatory mediators, cell signaling pathways are involved in GDM [308,309,310,311,312]. A high maternal blood glucose level acts as a major teratogenic agent by altering many normal signaling pathways involved in fetal development and organogenesis [313,314]. Several studies have shown that activation of phosphatidylinositol 3-kinase/protein kinase B (PI3K/AKT) and MAPK signaling pathways could promote the occurrence of hyperglycemia and IR [308,309]. The C-Jun N-terminal kinase (JNK) [315], JAK/STAT [310] and nuclear factor kappa B cells (NF-κB) pathways [312] are closely related to IR. The activity of JNK and insulin receptor substrate 1 (IRS-1) serine phosphorylation inhibits the occurrence of IR [316,317]. It has also been found that JAK/STAT could affect the secretion of proinflammatory cytokines, such as tumor necrosis factor alpha (TNF-α) and IL-6, thus mediating IR [303,310]. Also, the IKK/NF-κB signal pathway was involved in IR in DM [316,318] and the Toll-like receptor (TLR) pathway [314].

This intriguing notion that pro-inflammatory mediators, metabolic and transcriptional mediated pathways, are decisively involved in provoking the pathogenesis of IR has also been supported by many clinical observations where IR has been strongly correlated with systemic and/or local low-grade chronic inflammation. Nevertheless, the etiology of GDM and the activity of particular pro-inflammatory molecular pathways are still under debate.

### 9.6. Genetic Basis of Diabetes

A major goal in human genetics is to use natural variation to understand the consequences of altering each protein-coding gene in the genome. Twin and family studies provide evidence of genetic factors contributing to T1DM and T2DM risk and estimates of the familial aggregation of the disease based on risk in the relatives of an affected individual.

Twin studies have shown that concordance for T1DM and T2DM is greater for monozygotic twins, who share 100% of their genes. When a member of the pair has type 1 and type 2 diabetes (proband twin), the risk to the other twin is approximately 50%, suggesting that both genetic and nongenetic factors contribute to the risk [319,320,321]. Dizygotic twins and non-twin siblings share only 50% of their genes, and, accordingly, their concordance rate for T1DM and T2DM is lower [319,321,322,323]. It has been reported that individuals with T2DM-affected siblings are at a two- to threefold increased risk of developing T2DM compared with the general population [324]. Having one parent with diabetes increases the risk of T2DM by 30–40%, and having both parents with diabetes increases the risk by 70% [325].

Furthermore, there are specific forms of monogenic diabetes that suggest a genetic etiology. The genes known to cause monogenic diabetes include *GCK*, *HNF1A*, *HNF4A*, and *PDX1* [326,327]. Genetic factors may relate to the occurrence of mutations in genes (maturity onset diabetes of the young, MODY), as well as anti-insulin or anti-islet antibodies (latent autoimmune diabetes in adults, LADAs) [328]. Disorders in the functioning of insulin receptors are also causes of diabetes. A correlation between polymorphism in melatonin receptor genes (melatonin receptor 1B, MTNR1B), transcription factor 7-like 2 (TCF7L2) and tumor necrosis factor alpha (TNF-α) has been demonstrated [329,330]. Hayes and colleagues found an association between glucokinase regulator (GCKR), glucose-6-phosphatase catalytic subunit 2 (G6PC2), proprotein convertase subtilisin/kexin type 1 (PCSK1), protein phosphatase 1 regulatory subunit 3B (PPP1R3B) and MTNR1B and fasting glucose levels [331].

Genes and their variants within the human major histocompatibility complex (MHC) and the human leukocyte antigen (HLA) loci, including class I (HLA-A, -B, and -C) and class II (HLA-DR, -DQ, and -DP), account for ~50% of the genetic risk of T1DM. In addition to the MHC region, T1DM risk loci were initially identified through candidate gene and linkage studies, including variants in or near the INS genes (encoding insulin), PTPN22 (protein-altering structure), and CTLA4 and IL2RA (affecting T cell activation and differentiation) [332,333].

T2DM, as a polygenic condition, is well appreciated to be genetically determined by thousands of variants. Hundreds of independent SNPs have been associated with T2DM and glycemic traits using genome-wide association studies (GWASs), and their numbers continue to increase. Table 10 presents the 75 independent genetic loci associated with T2DM.

The major critics of genetic research on T2DM highlight the fact that the common variants with a relatively low effect size (an odds ratio between 1.10 and 1.40) explain only 10–15% of the T2DM heritability [335]. In addition, most of the variants are located in intergenic or intronic regions, where it is difficult to explain their functional consequences.

### 9.7. Epigenetics of Diabetes in Humans

**Figure 9:**
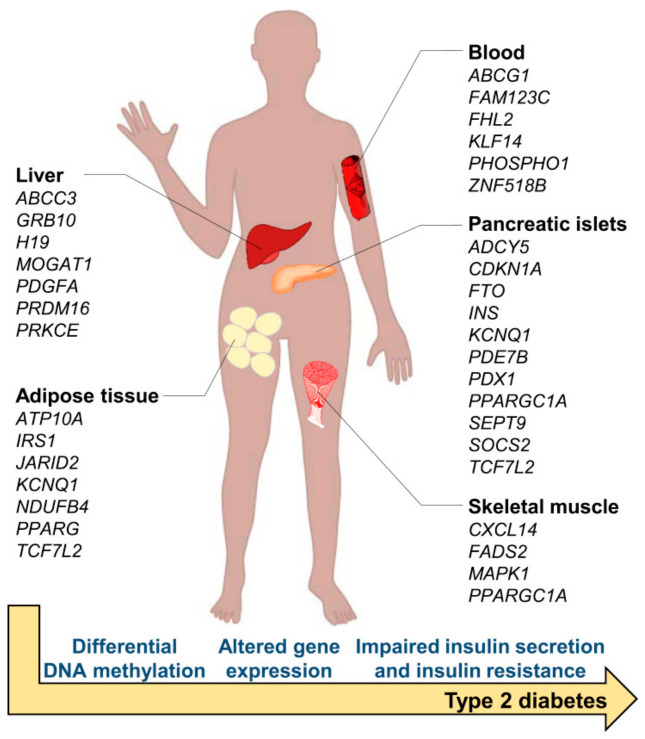
Epigenetics of type 2 diabetes in humans. Type 2 diabetes (T2DM) is associated with differential DNA methylation in human tissues. This figure has been taken from the article of Ling and Rönn [338] distributed under the terms and conditions of the Creative Commons Attribution License (CC BY-NC-ND/4.0/).

In addition to genetic factors, epigenetic changes in the genome have been implicated in diabetes. Epigenetics is one of the mechanisms linking environmental factors to altered genes. Epigenetic modifications such as increased DNA methylation, histone modifications and disturbed microRNA (miRNA) expression are associated with hyperglycemia and the development of diseases, including CHDs in humans [336]. Furthermore, since the epigenetic patterns are cell-specific, it is essential to study tissues of importance for a certain disease. The development of T2DM requires disturbances of multiple biological mechanisms in different organs, including the pancreas, liver, skeletal muscle and adipose tissue [337]. Figure 9 illustrates tissues and genes with alterations in DNA methylation observed in subjects with T2DM compared with non-diabetic controls.

Some of the genes shown in Figure 9 also show differential gene expression and have been shown to functionally affect diabetes-related phenotypes such as insulin secretion. DNA methylation of the genes *ABCG1*, *FAM123C*, *FHL2*, *KLF14*, *PHOSPHO1*, and *ZNF518B* indicated as being related to blood has been associated with future risk of T2DM in prospective cohorts [338].

DNA methylation of candidate genes for T2D, such as INS (encoding insulin), *PDX1*, *PPARGC1A* (encoding PGC1, which regulates insulin expression), and *GLP1R* (encoding the GLP-1 receptor), in human pancreatic islets from donors with T2D and non-diabetic controls was studied. Recent epigenome-wide association studies (EWASs) identified several DNA methylation markers associated with T2DM, fasting glucose and HbA1c levels [339]. Pancreatic islets from T2DM donors were found to have increased DNA methylation and decreased expression of the key genes associated with impaired insulin secretion. High glucose and glycated hemoglobin (HbA1c) levels increased DNA methylation of these genes [340,341,342,343,344].

On the other hand, Dayeh et al. [345] found *CDKN1A*, *PDE7B* and *SEPT9* genes with reduced DNA methylation and increased gene expression in pancreatic islets from donors with T2DM [345]. To mimic the situation of T2DM, these three genes were overexpressed in clonal β cells, which resulted in decreased glucose-stimulated insulin secretion.

In pancreatic islets, Dayeh et al. [345] also found differential DNA methylation of CpG sites annotated to several candidate genes for T2DM and obesity, as identified by genome-wide association studies (GWASs), such as *ADCY5*, *FTO*, *HHEX*, *IRS1*, *KCNQ1*, *PPARG*, and *TCF7L2* [345].

DNA methylation was also analyzed in human adipose tissue, liver, and skeletal muscle from subjects with T2DM and non-diabetic controls [346,347,348,349,350]. These studies identified numerous CpG sites with altered DNA methylation in target tissues from patients with T2DM, supporting the role of epigenetics in the pathogenesis of diabetes.

In addition to DNA methylation, studies have profiled changes in histone modification in T1DM. For example, researchers have measured levels of histone modifications, such as dimethylation and acetylation of H3 lysine 9 (H3K9me2 and H3K9ac), in lymphocytes and monocytes and found altered levels in T1DM compared to controls, including those measured in T1DM risk loci, e.g., CTLA4 and class II MHC [351,352]. Changes in histone modification levels, as well as histone acetyltransferase localization, have been observed in immune cells in disease-relevant environmental conditions, such as hyperglycemia [353,354]. Hyperglycemia alters epigenetic landscapes by increasing DNA methylation, suppressing histone deacetylation and perturbing microRNA (miRNA) expression.

There are many miRNAs that may have an impact on DM. They can inhibit insulin signaling, inhibit glucose uptake, promote insulin signaling, and reduce insulin secretion [265,355]. Deregulation of miRNA expression can directly impair β-cell function, leading to the development of T2DM [356]. More than 2600 miRNAs have been described within the human genome, and multiple miRNAs, including miR-200, miR-7, miR-184, miR-212/miR132 and miR-130a/b/miR-152, have been shown to be involved in the pathogenesis of T2DM [357].

Animal studies further support the hypothesis that epigenetic modifications in pancreatic islets may lead to altered gene expression, impaired insulin secretion and subsequently diabetes [358,359]. Studies using rodent models of maternal diabetes and single-cell transcriptomic and epigenetic profiling have identified several embryonic cardiac developmental processes and cardiac cell lineages that are impaired under hyperglycemic conditions [360,361].

Genetics and epigenetics improve the ability to predict T1DM and T2DM in individuals at risk, shedding light on the heterogeneity of the disease, and may provide evidence of underlying pathophysiological mechanisms that could be targeted for prevention and treatment.

## 10. Fetal Congenital Heart Defects as a Complication of Maternal Diabetes

Numerous epidemiological studies have demonstrated that matDM is a significant risk factor for structural birth defects in offspring, the most common anomalies being CHDs and neural tube defects [8,19,79]. However, the associations between maternal PGDM and GDM, as well as the risk of specific types of CHDs, remain under debate [362]. The offspring of mothers with preexisting diabetes (types 1 and 2) have an approximately 3-fold increased risk of any type of CHD, and GDM carries an approximately 1.5-fold increased CHD risk [38].

Clinical and epidemiological studies have established a strong link between maternal PGDM and an increased risk of neonatal CHDs and other adverse pregnancy outcomes [25]. “Diabetic embryopathies” affecting the cardiovascular and nervous system can be observed in the offspring of women with pregestational T1DM [363,364]. PGDM causes structural and morphological abnormalities in the developing embryonic heart, whereas GDM induces functional disorders in the fetal heart and long-term metabolic risks for both the woman and her offspring [365]. Nevertheless, the presence of PGDM carries a greater risk of complications in the newborn. This may be due to the presence of vascular changes in type 1 and 2 diabetes and its longer duration than that of GDM. However, the prevalence of offspring CHDs correlates with increased or poorly controlled maternal blood glucose levels, measured by glycated hemoglobin (HbA1c) [366]. In the case of PGDM (type 1 or 2), proper glycemic control and (HbA1c < 6.5%) in the pregestational period and in the first trimester of pregnancy significantly reduces the incidence of complications in the newborn [367,368].

A variety of cardiac malformations have been reported in the offspring of women with PGDM. Several meta-analyses conducted by different research groups have consistently identified specific CHD phenotypes occurring with an increased prevalence upon exposure to maternal hyperglycemia. These phenotypes include TGA, heterotaxia, VSDs, and ASDs [37,38]. While T1DM and T2DM have similar incidences of CHDs, T1DM is associated with a higher risk of conotruncal defects and AVSDs, whereas T2DM is more commonly associated with heterotaxia and left ventricular OFT obstructive abnormalities [39].

Population-based studies on the offspring of PGDM mothers demonstrated CHDs originating from an altered differentiation of the anterior SHF progenitor cells (PTA, TOF, DORV, HLHS, and VSDs) to be more frequent than other CHD types [23]. The above suggests that progenitors of the anterior SHF are more sensitive to maternal hyperglycemia than those of the posterior SHF [40,369]. Nevertheless, ASD cases in diabetic pregnancies have also been reported [23]. Although less prevalent, these ASDs suggest that maternal hyperglycemia impacts the patterning of the posterior SHF [37,40].

However, inconsistent results were retrieved from studies evaluating the association between gestational diabetes mellitus (GDM) and CHDs in offspring. Subsequent analysis according to the types of CHD showed that maternal GDM was associated with higher risks of ASDs, VSDs, and ToF [41]. To examine the independent associations between maternal exposure to diabetes during the periconceptional period and the occurrence of CHDs in offspring, odds ratios (ORs) and 95% confidence intervals (CIs) were calculated using a multivariate logistic regression model.

Chen et al. [42] conducted a systematic review and meta-analysis to assess the risk of CHDs and the specific phenotypes associated with matDM, including PGDM and GDM. Their study suggests that the risk of CHDs is significantly higher among mothers with PGDM than those with GDM. Additionally, this study suggested that matDM is significantly associated with most phenotypes of CHDs; of these, double outlet of the right ventricle (OR  =  10.89; 95% CI: 8.77–13.53), atrioventricular septal defects (OR  =  5.74; 95% CI: 3.20–10.27) and truncus arteriosus (OR  =  5.06; 95% CI: 2.65–9.65) were identified as the first three of the most common phenotypes of CHDs associated with matDM.

In a large prospective cohort study conducted between January 2011 and March 2014 in Japan, it was observed that matDM, including both PGDM and GDM, was associated with an increased risk of CHD in offspring: multivariable OR (95% CI) = 1.81 (1.40–2.33) for matDM, 2.39 (1.05–5.42) for PGDM and 1.77 (1.36–2.30) for GDM. A higher risk of offspring CHD was observed in pre-pregnancy BMI ≥ 25.0 kg/m^2^ (OR = 2.55, 95% CI: 1.74–3.75) than in pre-pregnancy BMI < 25.0 kg/m^2^ (OR = 1.49, 95% CI: 1.05–2.10, *p* for interaction = 0.04) [245].

A large population-based study using health care records in China reported the association between matDM and the risk of offspring CHD (OR = 1.80, 95% CI: 1.31–2.46) [370].

Another large population-based study of 48,249 patients with CHD that used data from the Texas Birth Defects Registry and state-wide vital records for deliveries demonstrated an association with offspring CHD, even after adjustment for race/ethnicity (34.0% White, 10.8% Black, 52.3% Hispanic, and 2.9% others), for maternal diabetes (PR = 1.93, 95% CI: 1.84–2.03), pre-pregnancy diabetes (PR = 3.24, 95% CI: 2.86–3.67), and GDM (PR = 1.49, 95% CI: 1.39–1.60) [38].

A Canadian study conducted on a population-based sample of nearly 2.3 million infants born in Canada between 2002 and 2010 also investigated the link between type 1 and type 2 matDM and specific CHD subtypes. The researchers found type 2 matDM to be associated with the highest risk of heterotaxy and left ventricular outflow tract obstructive malformations, while type 1 matDM carried the highest risk for conotruncal malformations and atrioventricular septal defects [39]. It is noteworthy that type 1 and type 2 matDM raised, although to lower levels, the risk of other types of CHD examined (right ventricular outflow tract obstructive malformations and atrial and ventricular septal defects).

Among births in Norway in 1994–2009, in women with PGDM (adjusted RR = 2.23, 95% CI: 1.39–3.59) or GDM (adjusted RR = 2.73, 95% CI: 1.53–4.85), giving birth to a large-for-gestational-age neonate was associated with a 2- to 3-fold increased risk of cardiac defects in offspring compared to neonates with a normal birth weight [59]. Other studies have reported a similar association between matDM, including PGDM and GDM, and the risk of CHD in offspring [23,59,371].

The above results emphasize the fact that, today, PGDM is an important modifiable risk factor for CHDs, and for other birth defects as well, in many populations around the world experiencing an increasing prevalence of pregnancies complicated by PGDM.

In a systematic review and meta-analysis of population-based studies of over 80 million participants, Tie-Ning Zhang et al. investigated the associations between maternal PGDM and GDM and CHDs [362]. The authors suggested a statistically significant increase in the risk of CHDs in offspring of women with PGDM (RR = 3.46, 95% CI: 2.77 to 4.32, I^2^ = 98.2%, *p* < 0.001). Similarly, maternal type 1 and type 2 diabetes were associated with increased risk of CHDs in offspring (type 1: RR = 3.75, 95% CI: 1.86 to 7.57, I^2^ = 99.1%, *p* < 0.001; type 2: RR = 3.15, 95% CI: 1.72 to 5.78, I^2^ = 93.6%, *p* < 0.001). Maternal PGDM was associated with an increased risk of all specific types of CHDs available for examination in the present study. The RRs of specific types of CHDs ranged from 2.23 (for hypoplastic left heart; 95% CI: 1.07 to 4.64, I^2^ = 64.0%, *p* = 0.040) to 12.16 (for truncus arteriosus; 95% CI: 7.52 to 19.68, I^2^ = 0%, *p* = 0.866). However, maternal GDM is associated with CHDs (RR = 1.50, 95% CI: 1.38 to 1.64, I^2^ = 81.2%, *p* < 0.001). With respect to specific types of CHDs, the authors found that the offspring of women with GDM were at an increased risk of heterotaxia (RR = 5.70, 95% CI: 1.09 to 29.92, I^2^ = 85.7%, *p* = 0.008), ToF (RR = 1.41, 95% CI: 1.20 to 1.66, I^2^ = 0%, *p* = 0.600), LVOTOs (RR = 1.67, 95% CI: 1.15 to 2.41, I^2^ = 50.0%, *p* = 0.112), CoA (RR = 1.50, 95% CI: 1.23 to 1.83, I^2^ = 35.4%, *p* = 0.213), RVOTOs (RR = 1.25, 95% CI: 1.03 to 1.53, I^2^ = 0%, *p* = 0.739), VSDs (RR = 1.31, 95% CI: 1.24 to 1.38, I^2^ = 0%, *p* = 0.960), and ASDs (RR = 1.45, 95% CI: 1.40 to 1.50, I^2^ = 0%, *p* = 0.426).

Table 11 presents an overview of the association between the 18 analyzed subtypes of congenital heart defects in offspring and maternal PGDM and GDM. The RRs of overall CHDs in the offspring of women with PGDM were higher than those in the offspring of women with GDM. This means that the risk of CHDs in offspring was higher in women with PGDM than in those with GDM [362]. In earlier studies, a similar result was observed with respect to the risk of occurrence of most CHD phenotypes in the offspring of women with PGDM [38,42] and of women with GDM [372,373,374,375].

Other population-based studies have consistently indicated a 3–5-fold higher prevalence of CHD among offspring when exposed to pre-existing matDM [23,38,376,377].

## 11. Obesity

### 11.1. Definition and Classification of Obesity

Obesity is a multifaceted disease widely regarded currently as the main risk condition for developing metabolic syndrome. The basis of this disorder is an imbalance between energy consumption and energy intake [378]. According to the WHO (World Health Organization) definition, obesity is the abnormal or excessive accumulation of body fat, leading to a deterioration in the patient’s health. Precise definitions of obesity are under debate. Obesity affects the patient’s quality and length of life. Obesity in pregnancy has an adverse effect on both fetal and neonatal outcomes, including increased risks of major congenital malformations [379]. The development of obesity is influenced by many factors, genetic, environmental, socioeconomic, psychological and hormonal, the coexistence of which can intensify the disease process. Excessive energy supply with food and insufficient energy expenditure are the main factors promoting obesity [378,380].

Many authors distinguish between primary and secondary obesity.

Primary obesity (simple, alimentary) is in 90% of cases associated with a disorder in the supply and expenditure of energy consumed by the body. In the case of the population of developmental age, a sustained positive energy balance increases the number and volume of adipocytes (hypertrophy and hyperplasia of fat cells), which predisposes to obesity in later years [378].

Secondary (symptomatic) obesity occurs in approximately 10% of patients. The secondary occurrence of excess body weight is caused by hormonal disorders (hypothyroidism, Cushing’s disease and syndrome, and growth hormone deficiencies) and genetic conditions (Down, Turner, Prader–Wille, Bardet–Biedl, Carpenter and Alstrom syndromes). Also, the use of glucocorticoids, antidepressants and antiepileptic drugs may affect the development of this type of obesity [381,382].

### 11.2. Classification of Obesity Based on Body Mass Index (BMI) According to WHO

According to the World Health Organization (WHO), BMI is most often used to assess the amount of adipose tissue in the body. Maternal BMI is categorized as underweight (BMI < 18.5 kg/m^2^); normal weight (BMI 18.5 to <25 kg/m^2^); overweight (BMI 25 to <30 kg/m^2^); and class I (BMI 30 to <35 kg/m^2^), class II (BMI 35 to <40 kg/m^2^), and class III (BMI ≥ 40 kg/m^2^) obesity [383,384] (Table 12).

### 11.3. Epidemiology of Obesity

Obesity is classed as a global epidemic by the World Health Organization (WHO) [383]. Although in the past it was observed only in high-income countries, the rate of obesity is now increasing in prevalence in many middle- and low-income countries. The dynamics of obesity growth in Europe are greater for men than for women (3.09% per year vs. 1.92% per year). With the growth rate remaining at the estimated level, in 2030 there will probably be more obese men (36.6%) than women (32.0%) in Europe [385].

In Europe, the proportion of obese subjects has increased over the past four decades four times among men and twice among women, and the number of diagnosed obesity cases ranges from 19% (Denmark) to 31% (Malta) [386]. The lowest rates of obesity were found among men in Portugal and Slovenia and among women in Denmark, and the highest rates of obesity among both men and women were found in Malta.

The lowest percentages of overweight people (34–46%) live in Romania, France, Russia and Ukraine, and the lowest percentages of overweight women (33–39%) live in Estonia, France, Italy and Denmark [387,388]. In 2030, the percentage of obese men is likely to exceed 40% in six countries: Bulgaria, Ireland, Romania, the United Kingdom, Malta and Hungary (up to 43.6%); and the percentage of obese women is likely to exceed 42.0% in two countries: the United Kingdom and Ireland [386].

Among the analyzed non-European countries, the highest numbers of obese men in 2016 were recorded in the USA, Kuwait and Canada, and the highest numbers of obese women were recorded in Kuwait, Saudi Arabia and the United Arab Emirates [386]. The percentage of obese people, especially among women, was much higher than the average in the European Union. The lowest number of obese people was recorded in the male population in Uganda, Bangladesh, Nepal and India (less than 3%) and in the female population (less than 5%) in Japan, Bangladesh, Nepal and India. Globally, a significantly smaller percentage of people with excess body weight is observed in Southeast Asia—India, Thailand, Vietnam, Indonesia, Korea, and China—although the obesity rate is also increasing in that region [389]. In North Africa, a 3-fold increase in overweight and obesity has been shown in 20 years, which is associated with lower-education societies [388,390]. An alarming increase in the percentage of people with a BMI > 25 kg/m^2^ is observed in developed countries. It is predicted that, in about 15 years, in the USA approximately 79% of people will have a BMI above 25 kg/m^2^, including about 50% with a BMI above 30 kg/m^2^ [391].

The increase in overweight and obesity in women occurs mainly at reproductive age, which is alarming because excessive pregestational weight is associated with the development of maternal pregnancy-related complications and has significant consequences on the short- and long-term health of offspring [392,393].

The prevalence of obesity varies by demographic factors, such as age, race and origin, educational attainment, and socioeconomic status [57,394].

### 11.4. Prevalence of Obesity Among Women of Reproductive Age

**Figure 10:**
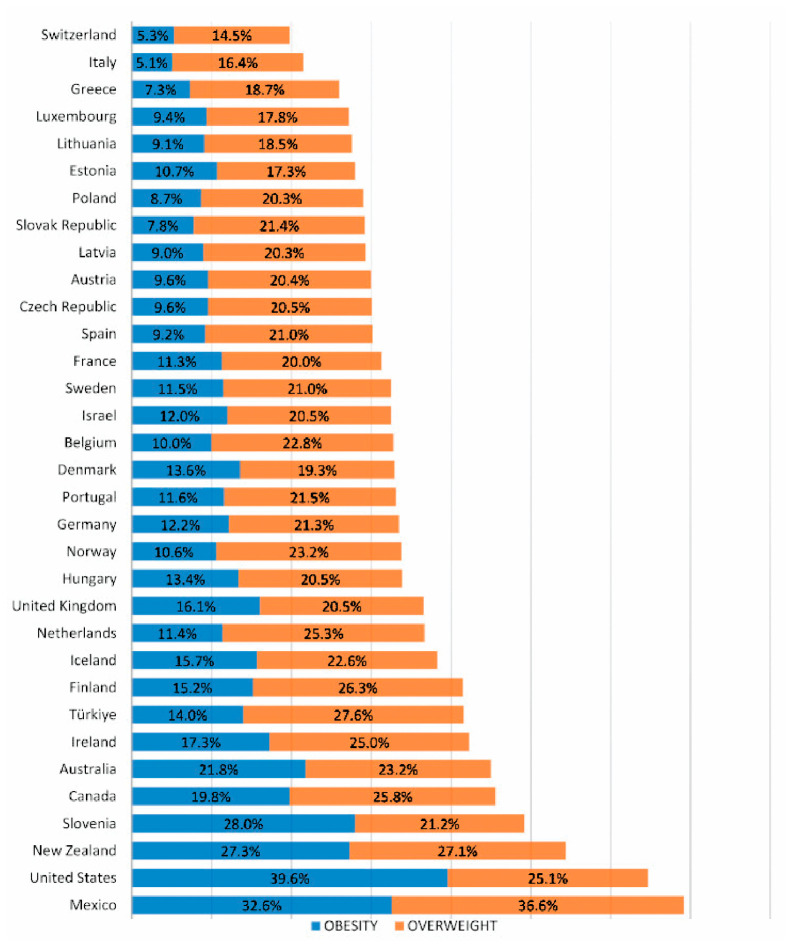
Prevalence of excessive weight in women. Prevalence data are shown only for member countries of the Organization for Economic Cooperation and Development. Women aged 18 to 44 were included in most countries. This figure has been taken from the article of Valencia-Ortega et al. [397] distributed under the terms and conditions of the Creative Commons Attribution License (CC BY-NC-ND/4.0).

Obesity among women of reproductive age is increasing worldwide and is now a common health problem in pregnancy [56,57,395,396]. Figure 10 presents data on the prevalence of excess body weight among women of reproductive age from the member countries of the Organization for Economic Cooperation and Development only.

There is evidence that overweight and obesity are prevalent among women of reproductive age, accounting for 40% to 60% in developed countries and 30% to 40% in developing countries. In European countries, the prevalence of obesity before pregnancy ranges from 7.8% to 25.6% [398,399]. In Sweden, from 1992 to 2014, the prevalence of early pregnancy obesity (BMI ≥ 30) increased from 6.0% to 12.9%, and the prevalence of obesity class III (BMI ≥ 40 kg/m^2^) has more than tripled [400].

In Australia, the prevalence of obesity (BMI 30+) increased from 7.1% in 1980 to 18.4% in 2000, and by 2000 22.6% of 25- to 34-year-old Australian women were overweight (BMI ≥ 20 to <30) and 12.4% were obese [401,402]. In the United States, the prevalence of early pregnancy obesity was nearly 3 in 10 women, with only 45% of mothers having a normal weight when becoming pregnant [403,404], while nearly 4% were underweight [405]. According to estimates, the overall prevalence of obesity in women aged between 20 and 39 years in the United States increased from 15% in 1976–1980 to 34% in 2008 [406,407,408].

He et al. [409] presented national estimates of the prevalence of overweight or obesity and underweight among reproductive-age women based on a national mega-survey conducted between 2010 and 2014 in rural China. They found that among reproductive-age women (20–49 years old), the prevalence of overweight or obesity was 16.5% (95% CI: 16.4%, 16.6%) and that of underweight was 7.8% (95% confidence interval [CI]: 7.7%, 7.9%) according to the WHO criteria. According to Chinese criteria, the overweight and obesity prevalence reached 24.8% (95% CI: 24.7%, 24.9%) for these women [409]. Moghimi-Dehkordi et al. reported that in Iran the overall prevalence of overweight and obesity was 34.1% and 15.4% and that their prevalence in the general Iranian population is moderately high [410].

Globally, it has been reported that the number of women aged 18 years and older with a BMI ≥ 35 doubled from approximately 50 million to 100 million between 2000 and 2010 [411]. It has been projected that by 2025 more than 21% of women in the world will be severely obese [412]. As suggested by linear time trend forecasts, 51% of the population will be obese by 2030. The model estimates a much lower obesity prevalence of 42% and severe obesity prevalence of 11% [413].

**Figure 11:**
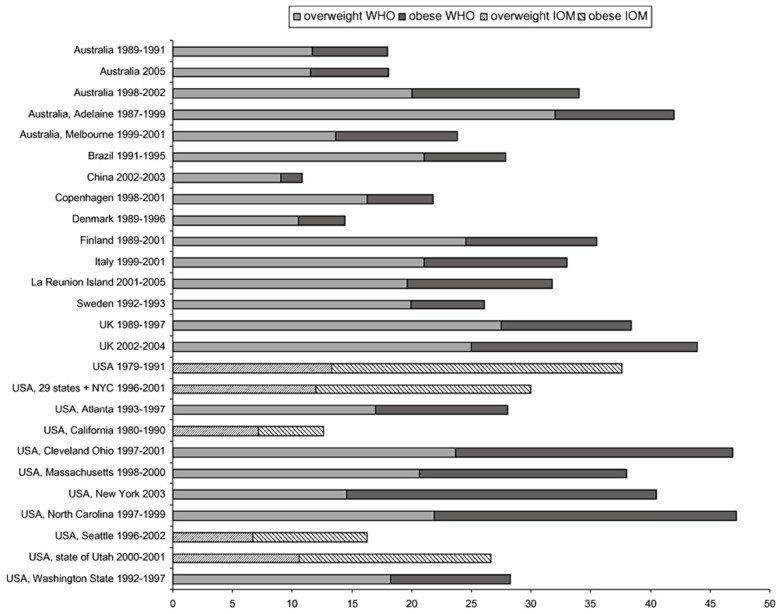
Prevalence (%) of overweight and obesity in pregnant women. IOM, Institute of Medicine; WHO, World Health Organization. This figure has been taken from the article of Guelinckx et al. [414] distributed under the terms and conditions of the Creative Commons Attribution License (CC BY-NC-ND/4.0).

**Figure 12:**
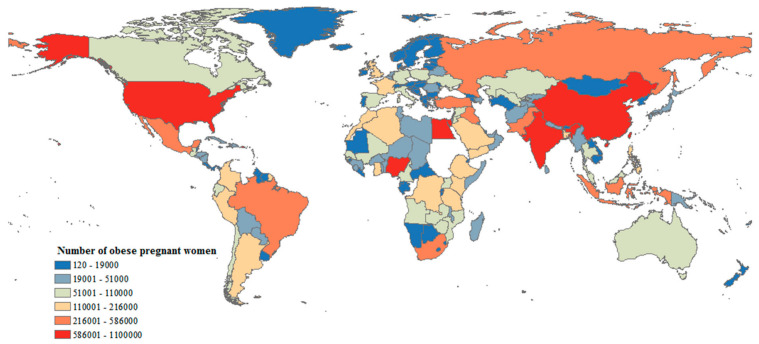
Obesity in pregnant women. The estimated distribution of obese pregnant women—the global perspective (184 countries, the time-related trend from 2005 to 2014). This figure has been taken from the article of Chen et al. [57] distributed under the terms and conditions of the Creative Commons Attribution License (CC BY-NC-ND/4.0).

Figure 11 summarizes studies reporting the prevalence of pre-pregnancy overweight and obesity among pregnant women from different countries [414]. This increasing trend is occurring in many countries Figure 12.

### 11.5. Pathogenesis of Obesity and Factors Influencing Its Development

The pathophysiology of obesity is not well understood. The development of obesity is influenced by many factors from the prenatal period to adulthood. These include environmental, hormonal, psychological, genetic and epigenetic factors, as well as changes in the gut microbiome [415,416,417,418]. All these factors, in turn, create a network of relationships which are the basis for the concept of the multifactorial pathogenesis of obesity and its comorbidities.

Among the hormonal factors, insulin, which activates lipogenesis, inhibits lipolysis and is involved in the differentiation of fat cells, playing a key role in the development of obesity [419]. Cortisol has an effect on fat accumulation and mobilization and inhibits the antilipolytic effect of insulin in adipocytes, especially in visceral adipose tissue. An important weight regulator is growth hormone. Its deficiency leads to an increase in the amount of subcutaneous fat, which in adults is deposited mainly in the trunk area. Untreated hypothyroidism can also be a cause of obesity, as low levels of T3 and T4 result in a slowdown in metabolism and the accumulation of fat and water. Gonadal steroids cause a change in the ratio of fat to lean body mass in girls and boys. During menopause, a sharp drop in estrogen levels causes an increase in visceral fat mass and an increase in insulin resistance. Progesterone helps to maintain normal estrogen levels and stabilizes blood glucose levels, while women with low progesterone levels may have fluctuations in insulin blood levels and problems with maintaining a healthy weight. Testosterone reduces fat mass in men and increases muscle mass during puberty; however, with age, low levels cause the accumulation of visceral fat. Excess androgens also play an important role in the development of visceral obesity [420]. The hyperandrogenic state is also associated with metabolic disorders in the course of polycystic ovary syndrome, contributing to the occurrence of complications ranging from obesity to insulin resistance [421].

Weight gain can also develop with long-term exposure to chronic stress, which is characterized by increased activity of the hypothalamic–pituitary–adrenal and sympathetic system axes [420]. Leptin affects receptors in the hypothalamus, inducing a feeling of satiety and giving a signal to stop eating. Leptin signal disorders due to too low levels or lack of sensitivity of the receptors to its action cause a lack of satiety and excessive food intake. A long-term increase in leptin levels in the blood can lead to leptin resistance, a condition in which leptin receptors stop recognizing it. Ghrelin, also referred to as the “hunger hormone”, together with leptin, is responsible for the balance between hunger and satiety [422]. During the fasting period, the endocrine cells of the stomach secrete ghrelin, which, like leptin, goes to the hypothalamus and connects to ghrelin receptors. Then the neuropeptide Y is activated, which stimulates the appetite and causes a feeling of hunger. After a meal, ghrelin levels drop and a feeling of satiety appears. Ghrelin negatively correlates with the concentrations of glucose and insulin in the blood. The most common hormonal syndromes associated with obesity include hypothyroidism, Cushing’s disease and syndrome, and polycystic ovary syndrome (PCOS) in women, as well as T2DM.

Deficiencies in most vitamins, especially fat-soluble vitamins, folic acid, vitamin B12 and vitamin C, have been found in obese subjects [423]. Vitamin D plays an intermediate but important role in carbohydrate and lipid metabolism [424,425]. A correlation between vitamin D receptor expression and obesity has been demonstrated [426], as well as the effect of vitamin D3 on the development of overweight and obesity [427]. Lipophilic vitamin D3 is stored in adipose tissue, among other areas, which reduces its concentration in the blood and its bioavailability [428]. The serum levels of 25(OH)D in obese individuals were shown to account for 71% of the values in non-obese subjects (*p* = 0.001) [429]. Too low levels of vitamin D3 in the blood coexisted with impaired insulin function and carbohydrate metabolism disorders. In a group of school-age children, it was observed that reduced blood levels of vitamin D3 were associated with an increase in BMI and the development of obesity (especially visceral obesity) and, regardless of the above correlation, with an increased risk of T2DM [430]. Studies have shown that vitamin A may also influence the development of obesity and obesity-related diseases, including insulin resistance, T2DM, and fatty liver and steatohepatitis, as well as cardiovascular diseases [431,432]. This vitamin is involved in lipid metabolism and acts on the storage and catabolism of fatty acids and adipocytes.

Some medications often contribute to weight gain, mainly through appetite stimulation. These include glucocorticoids, excessive insulin doses, sulfonylurea derivatives, progesterone, estrogens, cyproheptadine, some antiepileptic drugs (e.g., valproic acid), antidepressants (e.g., amitriptyline and mianserin) and neuroleptics (e.g., phenothiazine derivatives and olanzapine) [433]. Beta-blockers may also promote weight gain, possibly by inhibiting thermogenesis [434].

Based on the latest research, it has also been hypothesized that highly processed foods may induce addictive behaviors [435]. In a group of 625 people, a higher score on the Yale Food Addiction Scale (Yale Food Addiction Scale, YFAS) was associated with impulsive and compulsive food consumption, higher BMI, neuroticism, and stress [436]. Neuroanatomical correlations between obesity and symptoms of food addiction have also been demonstrated. Increased BMI values were accompanied by lower volumes of some areas of the cerebral cortex, mainly in the frontal lobes [436,437,438]. It has been found that structural differences in the frontal areas of the brain, genetically determined or induced by factors related to obesity, can exacerbate abnormal eating behaviors and lead to weight gain or decreased dietary success [439]. Women with overweight or obesity and food dependence according to the YFAS, compared to women who were only overweight or obese, showed different responses to food signals (highly and minimally processed) in the upper right frontal gyrus on fMRI [440]. In a similar group of obese people, impaired control of binge eating inhibition was found [441], and in cases of morbid obesity, lower binding of the dopamine D2/D3 receptor in the striatum was demonstrated [442]. An important positive correlation between symptoms of food addiction and higher activation of the basolateral part of the amygdala, which is associated with susceptibility to weight gain and promotion of appetite behavior, has been identified [440].

A growing number of studies support the concept of bidirectional signaling within the brain–gut–microbiome axis in the pathophysiology of obesity, mediated by metabolic, endocrine, neural and immune system mechanisms. An imbalance in the gut microbiota has been indicated as a potential factor leading to obesity [416,443,444,445]. The gut microbiota affects host metabolism and obesity through several pathways involving gut barrier integrity, production of metabolites affecting satiety and insulin resistance, and epigenetic factors, as well as metabolism of bile acids and subsequent changes in metabolic signaling [443,445]. Gut microbiota management has become a new method of treatment of obesity.

Psychological factors include disorders of the self-regulation mechanism, difficulties in coping with stress, and eating disorders. Psychological factors can lead to compulsive eating and excessive calorie consumption. Eating disorders such as bulimia and anorexia can contribute to obesity.

Another important reason for the development of obesity is limited physical activity, a sedentary lifestyle, passive rest, a reduction in time spent on and a reduction in physical exertion during various household chores, reduced work-related energy expenditure, and the absence or limitation of recreational physical activity.

### 11.6. Genetic Basis of the Development of Obesity

The genetics of obesity is a complex issue, as metabolism, appetite regulation, and fat accumulation can be affected by many different genes. Currently, it is estimated that a genetic basis is important in approximately 25–40% of cases of overweight [446]. It has been observed that obesity is twice as common in identical twins as in non-twin siblings, and the risk of its occurrence increases 4–5 times and 13 times when one or both parents are found to be overweight [447]. The inheritance of the vast majority of cases of obesity is very complex and polygenic in nature [448]. Single-gene obesity is rarely observed (about 200 such cases have been described, e.g., mutations in the leptin gene, leptin receptor, and melanocortin receptor 4), as is obesity that is part of a genetic syndrome (e.g., Prader–Willi syndrome or Bardet–Biedel syndrome) [382,449].

The recent advent of sophisticated genetic and genomic methods has led to the identification of numerous genes associated with obesity and diabetes mellitus. Mutations associated with overweight may occur in genes regulating appetite and food intake, metabolism or adipocyte maturation. One of the most commonly studied genes associated with obesity is the fat mass and obesity-associated (*FTO*) gene. The *FTO* gene is located on chromosome 16, and its various variants can affect appetite regulation, overall metabolism, and how fat is stored [450]. In particular, two studies have demonstrated that the variants rs9939609 and rs9930506 in the first intron of the FTO gene are significantly associated with BMI, thus suggesting that the presence of these variants may increase the risk of obesity in both adults and children [451,452].

In genome-wide association studies (GWASs), more than thirty sites strongly associated with obesity diagnosed by BMI have been identified [453,454]. These loci of susceptibility to obesity are found in the *GNPDA2*, *SH2B1*, *TMEM18*, *MTCH2*, *CDKAL1*, *FAIM2*, and *MC4R* genes, among others. There are also other genes that affect body weight, e.g., melanocortin 4 receptor (MC4R). The obesity risk alleles in MC4R have been shown to be associated with increased calorie intake [455,456] and the SH2B1 alleles with increased fat intake [457]. Kang et al. [458] found that loci near TMEM18 (rs6548238), CDKAL1 (rs7754840), and FAIM2 (rs7138803) may be associated with obesity-related indicators, and loci near TMEM18 (rs6548238) and FAIM2 (rs7138803) may increase susceptibility to the coexistence of obesity and T2DM. Recent GWASs include the identification of novel loci associated with obesity (SEMA-4D, PRKCA, and WARS2) [459]. The polymorphism of the Pro12Ala isoform PPARγ2 (peroxisome proliferator activated receptor, PPAR) has been shown to be manifested in increased BMI, hip circumference, lipid and carbohydrate metabolism disorders, leptin and adiponectin concentrations, and higher blood pressure values [456].

Since there are several publications reporting the critical pathogenic role of pro-inflammatory cytokines in obesity, further genetic association studies have focused on the impact of variants in genes encoding for these molecules in obesity [460]. Increased production and secretion of a wide range of inflammatory molecules, including tumor necrosis factor-alpha (TNF-a) and interleukin-6 (IL-6), has been reported in white adipose tissue in obesity [461]. TNF-a was found to be involved in lipid metabolism leading to hypertriglyceridemia as a result of decreased lipoprotein lipase activity and increased hepatic de novo synthesis of fatty acids, while IL-6 was found to be mainly associated with liver and adipose tissue inflammation [462]. The rs1800629 variant in the TNF-a gene, associated with increased expression of the cytokine in adipose tissue, has been reported to occur more frequently in obese than in lean subjects [463,464].

A study investigating genetic influences on extreme obesity indicated several loci that are linked to either the circadian rhythm of food consumption or hypothalamic signaling related to food intake [465,466]. Growing evidence shows that genetic variation (typically SNPs) can also affect epigenetic profiles independently of, or in combination with, environmental factors. Over the next decade, we can anticipate huge advances in capitalization on the biological promise of human genetics for metabolic diseases, delivering causal pathways and mechanisms for these diseases. These advances will provide a platform for improvements in obesity and diabetes diagnosis, prevention and management.

### 11.7. The Influence of Epigenetics on the Development of Obesity

**Figure 13:**
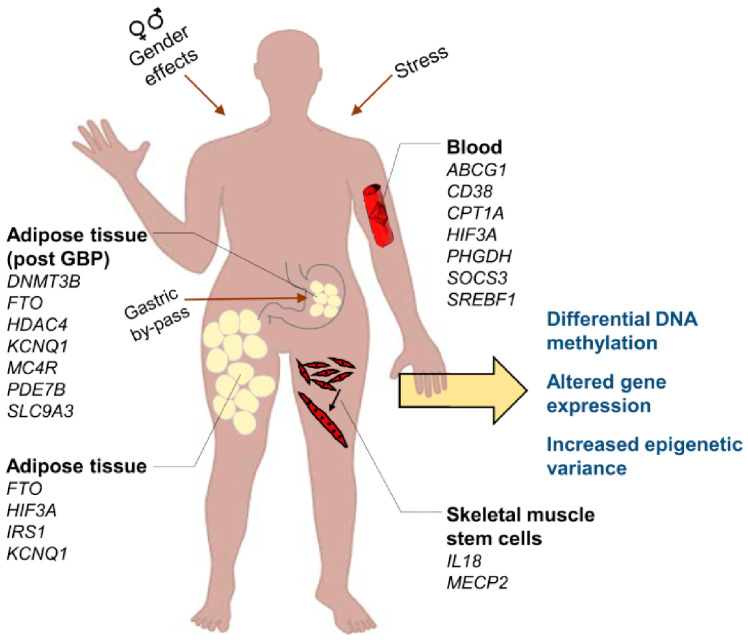
Epigenetics of obesity. Obesity is associated with differential DNA methylation and increased epigenetic variability. GBP, gastric bypass. This figure has been taken from the article of Ling and Rönn [338] distributed under the terms and conditions of the Creative Commons Attribution License (CC BY-NC-ND/4.0/).

Epigenetic changes in the human genome are defined as heritable regulatory mechanisms of gene expression overlaying the information enclosed in the DNA sequence. Obesity and related phenotypes induce epigenetic dysregulation, seen as increased variability in DNA methylation. DNA methylation, one of the most frequent and well-characterized epigenetic modifications, reflects at the molecular level a wide range of environmental exposures and genetic influences [467]. By stabilizing chromatin structure and altering gene expression, DNA methylation may influence an individual’s susceptibility to obesity and to the development of adiposity-related diseases such as diabetes, dyslipidemia, cardiovascular disease and CHDs [468]. It has been reported that DNA sequence variants or mutations affecting enzymes responsible for modifying or sensing epigenetic marks have been identified in patients with CHDs [469]. Furthermore, since the epigenetic patterns are cell-specific, it is essential to study tissues of importance for a certain disease, e.g., blood, adipose tissue and skeletal muscle stem cells for obesity. Increased methylation with increased BMI was confirmed in blood with altered expression of the *ABCG1*, *CD38*, *CPT1A*, *HIF3A*, *PHGDH*, *SOCS3*, and *SREBF1* genes [470,471,472,473,474,475]. An interaction between stress and DNA methylation of one CpG site in *SOCS3* in whole blood and its influence on obesity was also identified (Figure 13) [471,475].

Several studies have also investigated the association between DNA methylation and obesity-related traits and confirmed the altered gene expression of *HIF3A*, *FTO*, *IRS1*, and *KCNQ1* in subcutaneous adipose tissue [474,476] and *FTO*, *DNMT3B*, *HDAC4*, *KCNQ1*, *MC4R*, *PDE7B*, and *SLC9A3* in adipose tissue after gastric bypass associated with weight loss [477,478], as well as IL18 and *MECP2* in skeletal muscle stem cells (Figure 13) [479].

Figure 13 illustrates tissues and genes associated with observed alterations in DNA methylation due to obesity and related phenotypes (BMI and waist circumference), some of which are also associated with gene expression.

It has been suggested that perturbation of hypoxia-inducible transcription factor pathways could also have an important role in the response to increased weight in humans [474,480,481]. Increased BMI in adults of European origin has been shown to be associated with increased methylation at the *HIF3A* locus in blood cells and in adipose tissue. The DNA methylation data from human adipose tissue supports a role for epigenetics in the pathogenesis of the disease. Importantly, the DNA methylation sites associated with obesity predicted future risk of T2DM, which is a major clinical condition associated with obesity [481].

A high-fat maternal diet, especially in conjunction with maternal diabetes, has been demonstrated to induce lipotoxic effects in the developing offspring’s heart [482]. Together, these studies support an impact of BMI on epigenetic variation in candidate genes for both obesity and T2DM, which seems to affect gene expression and metabolism [338,468].

In addition to DNA methylation, studies have profiled changes in histone modification in obesity. Blin et al. found by measuring histone marks and global DNA methylation levels that maternal exposure to a high-fat diet induced long-term derepressive chromatin marks in the offspring’s heart. There is evidence suggesting that maternal high-fat exposure upregulates cardiac developing genes, such as isl lim homeobox 1 (*ISL1*) and six homeobox 1 (*SIX1*), by decreasing di- and trimethylated histone H3 and ubiquitinated histone H2A levels [483]. As demonstrated by chromatin immunoprecipitation sequencing studies, the offspring of obese mothers show a differential peak distribution on gene promoters related to acetylation of lysine 9 and 14 and trimethylation of lysine 4 and 27 in histone H3. Many of these genes are associated with metabolic processes and cardiac disease susceptibility [484].

Non-coding RNAs, especially microRNAs (miRNAs), are another class of epigenetic markers that have been linked to obesity and risk for other metabolic diseases [485]. These miRNAs can affect mRNA stability and degradation by binding anywhere along the length of the mRNA transcript [486,487]. Like genetic variants, several loci that correspond to miRNAs have been identified. Kunej et al. [488] identified 1736 genomic loci associated with obesity, of which 221 correspond to miRNAs. Several studies have also found these miRNAs to be correlated with diet and lifestyle [489,490], the key ones being the miR-17/20/93, 21-590-5p, 200 b/c, 221/222, let-7/miR-98, and miR-203 families of miRNAs. In a study conducted in children, a sex-specific association with obesity was found for the following miRNAs: 26 b-3p, hsa-576-5p, hsa-31-5p, hsa-10b-5p, and hsa-31-5p [489]. The understanding of the human epigenome and its interactions with the environment, particularly diet, may provide greater insight into the pathogenesis of obesity and help develop personalized nutritional interventions.

Epigenome-wide association studies (EWASs) using tissues would provide a comprehensive insight into the etiology of the disease; however, access to such samples is not possible on a large scale. That is why most EWASs have been conducted using whole blood [491]. Although more evidence is needed, literature reports strongly suggest that the analysis of the epigenetic architecture at the interface between gene expression and the epigenetic environment could be relevant for a better understanding of obesity and its associated comorbidities.

Understanding the role of diet-induced early epigenetic cues in the pathogenesis of disease is essential to halting its progression before the onset of symptoms and could be a key to reducing the global burden of metabolic diseases.

### 11.8. Mechanisms Influencing the Occurrence of CHDs in the Offspring of Obese Mothers

Maternal pre-pregnancy obesity is known to be associated with increased risk for gestational diabetes mellitus, and it is likely that some of the effect in obese individuals may be mediated by glycemic dysregulation [492]. In addition to glycemic dysregulation, a wide range of metabolic and endocrine abnormalities are present in obese subjects. Obesity is associated with hyperinsulinemia and insulin resistance [493,494], dyslipidemia [493,495], and oxidative stress [495,496,497,498]. Obesity is associated closely with a chronic low-grade inflammatory condition, which can give rise to the generation of reactive oxygen species, ultimately exerting detrimental effects on cells and tissues. Exposure to oxidative stress during fetal development can interfere with normal heart development and lead to CHD. The imbalance between reactive oxygen species (ROS) production and antioxidant defense mechanisms can lead to significant cellular and molecular alterations, contributing to the formation of structural anomalies. One of the primary mechanisms by which oxidative stress contributes to CHDs is through direct damage to the cellular components. Elevated ROS levels can induce lipid peroxidation, leading to membrane damage and altered fluidity, which compromises cell integrity and function. Additionally, ROS can modify proteins through oxidation, impairing their function and disrupting signaling pathways essential for cardiac development. Oxidative modification of transcription factors can alter gene expression, resulting in impaired cellular differentiation and growth. Oxidative stress can affect the key signaling pathways involved in cardiac morphogenesis. The development of the heart is regulated by various signaling cascades, including the Wnt/β-catenin pathway, transforming growth factor-beta (TGF-β) signaling, and the fibroblast growth factor (FGF) signaling pathway. Oxidative stress can interfere with these pathways, leading to abnormal cell proliferation, apoptosis, and improper cardiac tissue remodeling. Disruption of these signaling mechanisms during critical developmental windows can result in structural heart defects, highlighting the importance of maintaining redox balance for proper cardiac formation [496,497].

There is an increasing recognition that structural abnormalities and functional changes in the placenta can have deleterious effects on the fetal heart and development which can lead to CHD. The fetal heart and the placenta are directly linked because they develop concurrently with shared regulatory and signaling pathways. The mechanisms underlying this placental–fetal axis of interaction potentially include genetic factors, oxidative stress, chronic hypoxia, and/or angiogenic imbalance and nutrient transfer from the mother. Chronic hypoxia alters blood flow and affects the production of growth factors, leading to abnormal development of cardiomyocytes and, consequently, structural defects. Hemodynamic changes (an increase in cardiac afterload) can lead to abnormal development of the heart chambers and, in extreme cases, their underdevelopment. Disorders in signaling pathways (e.g., abnormal expression of the *HOXA13* genes) impair heart growth and vascular development. Defects of early placentation, such as abnormal placental implantation and incomplete remodeling of the spiral arteries, result in reduced uterine–placental flow, which induces chronic stress for the developing heart. Research also suggests that in some cases heart defects and placental pathologies result from the same genetic defects (placental–heart axis), which makes both structures susceptible to damage at the same time [498].

Maternal obesity can directly influence the functionality of the placenta, which has been identified as a contributing factor to changes in the development of fetal organs [499]. Obese women exhibit inefficient placental blood flow and compromised delivery of oxygen and nutritional factors that are important for fetal cardiac development [500,501]. Placental villous tissues from overweight and obese women were found to have a 6- and 14-fold increase in reactive oxygen species production, respectively [502]. Additionally, during pregnancy, women with obesity frequently exhibit decreased levels of circulating adiponectin [503], which has been linked to the development of placental insulin resistance [504] and increased placental nutrient transfer [505]. This is particularly significant because cardiac development occurs primarily during the first trimester, a period in which the fetus is unable to regulate glucose.

**Figure 14:**
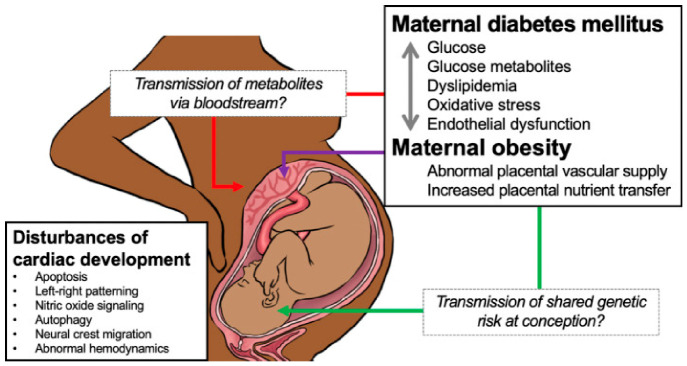
Potential mechanisms for transmission of maternal metabolic risk for congenital heart disease (CHD) in the fetus. Illustration of potential mechanisms of transmission of maternal factors during pregnancy influencing risk for CHD in offspring. Maternal diabetes mellitus and obesity share a variety of intermediate phenotypes (bidirectional gray arrow), which could be transmissible from mother to fetus in the blood across the placenta (red arrow) or transmitted genetically at the time of conception by pleiotropic variants, conferring risk for both metabolic phenotypes and CHD (green arrow). Specific differences in placental function related to maternal obesity may also contribute to risk (purple arrow). This figure has been taken from the article of Helle and Priest [56] distributed under the terms of the Creative Commons Attribution License (CC BY-NC-ND 4.0).

Epidemiological studies demonstrate that maternal obesity and maternal diabetes are often grouped together and considered on the same spectrum because they are associated with similar mechanisms through which they influence the development of a range of complex CHDs in offspring [30,31,56]. However, the precise mechanism by which maternal obesity and maternal diabetes influence crucial stages of fetal cardiac development remains largely unexplored and is hypothesized to be multifactorial [79]. The significant phenotypic overlap between diabetes mellitus, obesity, and cardiometabolic risk is complex; it has not been established yet which of these factors is responsible for risk to the fetus when present in the mother during early pregnancy. Figure 14 presents experimental models suggesting a variety of potential mechanisms by which maternal metabolic factors may disturb development of the heart, which occurs early in pregnancy during the first trimester.

Experimental models suggest multiple potential mechanisms by which maternal metabolic factors may disrupt cardiac development during the first trimester of pregnancy [9].

### 11.9. Congenital Defects of the Fetal Heart as a Complication of Maternal Obesity

Numerous epidemiological studies have demonstrated that maternal obesity during pre-pregnancy and pregnancy is associated with increased risks of severe fetal complications, heart defects (conotruncal heart defects) and malformations (neural tube defects, orofacial clefts, limb reduction defects, and urinary tract defects) [19,31,32,506,507,508]. The severity of CHDs varies but is often accompanied by increased mortality and long-term morbidity risks [214].

The increased risk associated with maternal obesity includes a wide range of different heart defects, including transposition of the great arteries, tetralogy of Fallot, septal defects, aortic arch defects, persistent ductus arteriosus, conotruncal defects, left ventricular outflow tract obstruction defects, right ventricular outflow tract defects and univentricular heart (UVH) [21,30,31].

Among numerous reports on associations of CHD phenotypes in infants born to obese mothers, the results of cohort studies and meta-analyses based on large populations of infants born in Nordic countries (Sweden, Denmark, Finland, Iceland and Norway) and non-Nordic countries [19,31,509,510] are particularly interesting. The authors calculated adjusted prevalence rate ratios (PRRs) with 95% confidence intervals (CIs) using a multivariate logistic regression model for each group of congenital heart defects in newborns in relation to the severity of maternal overweight and obesity.

**Figure 15:**
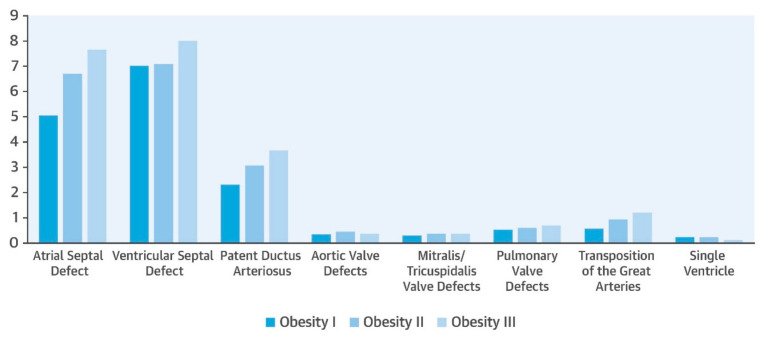
Maternal obesity and the risk of specific heart defects. This figure has been taken from the article of Persson et al. [31] distributed under the terms and conditions of the Creative Commons Attribution License (CC BY-NC-ND/4.0).

In the largest nationwide Swedish cohort study to date, including more than 2 million infants born between 1992 and 2012 to severely obese mothers without pregestational diabetes, the authors used the European Surveillance of Congenital Heart Malformations to classify CHDs and examined multiple CHD phenotypes [31]. This study showed that the PRR of aortic arch defects and TGA were increased in the neonates born to these mothers. In aortic arch defects, the adjusted PRR was 30% in offspring born to mothers with class I obesity, and it almost doubled in newborns to mothers with class III obesity. In the case of TGA, although this study showed an association with the more severe obesity category (class II obesity), it did not show evidence of a dose–effect relationship. In the case of ASDs and PDA, PRR prevalence in newborns also increased with increasing maternal BMI. For ASDs, the adjusted PRRs were 1.08 (95% CI: 1.02 to 1.14) and 1.65 (95% CI: 1.34 to 2.03) in the offspring of overweight mothers and the offspring of mothers with class III obesity, respectively. For PDA in term infants, the corresponding PRRs were 1.16 (95% CI: 1.06 to 1.27) and 2.32 (95% CI: 1.73 to 3.12), respectively. It was also observed that in the case of tetralogy of Fallot, atrioventricular septal defects, ventricular septal defects, aortic valve defects, right ventricular defects or univentricular heart, there was no clear correlation with maternal BMI. The PRRs of mitral–tricuspid valve and pulmonary valve defects increased with the severity of obesity but were not increased in the adjusted models. However, in the offspring of mothers with class I obesity, the risk of pulmonary valve defects was increased (Figure 15) [31].

The patterns of dose–effect relationships for maternal obesity severity and prevalence of ASDs and aortic arch defects found in the study by Persson et al. [31] are generally consistent with those observed in previous studies. For TGA, the study by Persson et al. [31] found associations that were in contrast to the results of the meta-analysis of maternal obesity and TGA by Cai et al. [511], who found no evidence of an association between TGA and any of the categories of maternal obesity, the sample size being larger than that reported in the study by Persson et al. [31]. Also, in the case of VSDs, for which no evidence of association with any obesity category was shown, the results are inconsistent with previous reports. Therefore, more studies are needed to explain these new observations. Although some studies reported no increased risk for CTDs, other studies reported risk elevations for TGA and defects of the great vessels and truncus arteriosus [33,34,35]. Waller et al. [33] described an elevated OR for TGA and great arteries defects, but not for the defects of septal closure. Queisser-Luft et al. [34], in their study, reported elevated ORs among mothers with a BMI > 30 for some heart anomalies, including truncus arteriosus and TGA.

In another study on a Swedish population-based cohort conducted from 2001 to 2014, it was observed that risks of congenital heart defects also progressively increased with maternal BMI (women with both pregestational diabetes and gestational diabetes were excluded) [31,56]. In this study, the adjusted risk ratios for CHDs by maternal BMI were 1.05 (95% CI: 1.01–1.08) for mothers who were overweight, 1.15 (1.09–1.20) for mothers with class I obesity, 1.26 (1.16–1.37) for mothers with class II obesity, and 1.44 (1.27–1.63) for mothers with class III obesity [31]. This is consistent with findings from a meta-analysis which also reported dose–response associations between the severity of maternal overweight and obesity and the overall risk of congenital heart defects, as well as risks of a few specific heart defects [511,512]. However, a Mendelian randomization study did not demonstrate a causal association between maternal BMI and the incidence of CHDs in the offspring [36].

Among all children born between 2006 and 2016 in Finland, maternal overweight has been found to be associated with increased odds of LVOTOs (OR = 1.28 [95% CI: 1.10–1.49]) compared with normal maternal BMI. Maternal obesity was associated with increased odds of complex defects (OR = 2.70 [95% CI: 1.14–6.43]), LVOTOs (OR = 1.24 [95% CI: 1.03–1.50]), and RVOTOs (OR = 1.31 [95% CI: 1.09–1.58]) in comparison with normal maternal BMI. Finally, maternal overweight was associated with lower odds of VSDs in offspring (OR = 0.92 [95% CI: 0.86–0.98]), and maternal underweight was associated with increased odds of pulmonary venous anomalies (OR = 6.75 [95% CI: 2.43–18.77]) compared with normal maternal BMI [19].

In a nationwide study conducted in Denmark between 2008 and 2018, 5442 out of the 547,105 pregnancies included in the cohort had CHDs (1.0%). The risk of CHDs became gradually higher with higher maternal BMI: for BMI 25–29.9 kg/m^2^, the adjusted odds ratio (aOR) was 1.17 (95% CI: 1.10–1.26); for BMI 30–34.9 kg/m^2^, the aOR was 1.21 (95% CI: 1.09–1.33); for BMI 35–39.9 kg/m^2^, the aOR was 1.29 (95% CI: 1.11–1.50); and for BMI ≥ 40 kg/m^2^, the aOR was 1.85 (95% CI: 1.54–2.21). The same pattern was observed in the severe CHD subgroup.

The proportions of CHD cases with one of the five specific CHD (UVH, TGA, AVSD, CoA and ToF) diagnoses was as follows: UVH: 4.2%; TGA: 2.8%; AVSDs: 4.2%; CoA: 4.1%; and ToF: 2.1%. No significant associations with maternal BMI were seen for UVH, TGA, CoA or ToF. However, maternal BMI ≥ 30 kg/m^2^ was positively correlated with a risk of AVSDs in offspring (OR = 1.67, 95% CI: 1.13–2.44) [510].

The authors also demonstrated a significant association between interpregnancy maternal weight gain ≥2 BMI units and higher risk of fetal CHDs in the second pregnancy (BMI 2 to <4 kg/m^2^: aOR = 1.29, 95% CI: 1.09–1.53; BMI ≥ 4 kg/m^2^: aOR = 1.36, 95% CI: 1.08–1.68) [510].

In a large US cohort study, obese and overweight women were more likely than normal-weight women to deliver an infant with any CHD, and obese women (BMI ≥ 30 kg/m^2^) had higher odds of having an infant with CTDs (OR = 1.34, 95% CI: 1.04–1.72), ASD (OR = 1.22, 95% CI: 1.04–1.43), or VSD (OR = 1.38, 95% CI: 1.06–1.79) [30].

Another population-based study conducted in New York State between 1993 and 2003 showed that maternal obesity increased the risk of ASDs, hypoplastic left heart syndrome, AS, PS and ToF. In all the women with obesity, there was a higher risk of having children with a CHD than in normal-weight women (OR = 1.15; 95% CI: 1.07, 1.23; *p*, 0.0001). On the other hand, in overweight women, no increased congenital defect risk to their newborns was observed [513]. Also, according to the findings of a recent meta-analysis, overweight women, as a group, were not at a significantly increased risk of any CHD or individual defects [507].

There are limited Australian data on the risk of birth defects associated with maternal pre-pregnancy obesity. A small case–control study conducted between September 1997 and March 2000 in western Australia provides evidence of an increased risk of congenital CTDs in newborns associated with maternal pre-pregnancy obesity [402]. Another Australian study we found on obesity and birth defects was that of Callaway et al. from Queensland, and they observed an overall increase in birth defects of 58%, but individual birth defects were not investigated [514]. These findings are supported by similar, statistically significant and more precise results from other overseas authors.

An observational study in China involving 1206 fetuses with congenital heart defects and 1112 fetuses without defects was conducted. The research showed an increase in congenital heart defects in underweight mothers compared to mothers with overweight or obesity. The lack of correlation between maternal obesity and congenital heart defects was attributed to the difference in the prevalence of obesity between the West and the East. A small number of overweight or obese mothers (BMI ≥ 24.0 kg/m^2^) took part in the study [515]. This explains why the result was inconclusive in comparison with the results from other studies conducted in Europe or America. Ghaderian et al. [516], examining a population of Iranian women in 2011–2012, also suggested that there may be no relation between maternal BMI and having a child with a congenital heart defect and that the most frequent congenital heart defects were VSDs (39%), PDA (11%), AVSDs (10%), PS (9.1%), and ASDs (8.5%) [516]. The results of recent meta-analyses by other authors suggest a moderate association between maternal obesity (BMI ≥ 30 kg/m^2^) and CHDs in offspring, with an odds ratio (OR) of 1.2 (95% CI: 1.1–1.2) [512] or 1.3 (95% CI: 1.2–1.4) [517]. A recent systematic review on the topic demonstrated great heterogeneity among the studies concerning the design, exposure definition, outcome definition, and choice of covariates, and only the populations of Northern European or Chinese descent were examined to a reasonable extent [21,510].

Liu et al. [91] conducted a large meta-analysis that included 6 cohort studies and 13 case–control studies conducted in North America, Europe, Oceania and Asia. Of the included studies, 9 reported that maternal obesity significantly increased the risk of CHDs in infants and 10 reported that there was no significant association between increased maternal BMI and increased CHD risk in the offspring. Subgroup analysis by study design showed that the significant association between maternal overweight and increased risk of infants with CHDs existed only in case–control studies, while the significant association between maternal obesity and increased risk of infants with CHDs existed in both cohort studies and case–control studies. A dose–response meta-analysis showed that each 5 kg/m^2^ increase in maternal BMI is accompanied by a 7% increment in CHD risk in infants. This meta-analysis discovered a risk increase of 8% for infants with CHDs in the maternal overweight group and an 23% risk increase in the maternal obesity group compared with the mothers with normal weight [91]. These findings are similar to those of a meta-analysis by Cai et al., who examined the association between maternal BMI and CHDs in offspring and reported a similar summary for overweight and obese individuals [511].

Salmeri et al. [32], in a meta-analysis conducted up to April 2023 that included 31 studies with 4,861,693 patients and 86,136 cases of CHDs, demonstrated the association of obesity with a 1.5-fold increase in the risk of severe CHD (pooled OR = 1.48 (95% CI: 1.03–2.13). Severe obesity was associated with an even higher risk, with 1.8-times higher odds compared with the reference group for specific subtypes of CHDs. All increased BMI categories were associated with a significantly higher risk of CTDs, and, in particular, the severe obesity category was associated with an elevated risk of both TGA (pooled OR = 1.39 (95% CI: 1.10–1.74)) and, to an even greater extent, ToF (pooled OR = 1.72 (95% CI: 1.38–2.16).

Increased BMI categories were also associated with a significantly higher risk of LVOTOs. In particular, all of these categories were associated with an elevated risk of HLHS. None of the BMI categories was associated with an increased risk of AS. All categories of increased BMI were associated with a significantly higher risk of RVOTOs, with the pooled OR escalating progressively with the increase in BMI. It is noteworthy that the risk of PS increased for all BMI categories, again with a progressive effect.

Compared with the reference category, women with moderate and severe obesity were at a significantly higher overall risk of septal defects (SDs). Specifically, the risk of VSDs and AVSDs increased only in the offspring of women with severe obesity, whereas the risk of ASDs was elevated in all categories of increased BMI [32].

The above studies express different opinions and are often difficult to compare. Therefore, the results from these meta-analyses must be interpreted with caution. The conflicting results of these studies suggest that the effect of maternal obesity on the offspring’s heart remains uncertain.

Table 13 presents an overview of associations between the 13 analyzed subtypes of congenital heart defects in offspring in relation to maternal exposure to metabolic disorders (obesity, diabetes, hypertension, preeclampsia, dyslipidemia, or MetS) [21]. The authors demonstrated that some disorders increased the risk of CHDs marginally, whereas pregestational diabetes and early-onset preeclampsia were strongly associated with CHDs, without consistent differences between CHD subtypes. The results are presented for maternal obesity (BMI ≥ 30 kg/m^2^).

Since prenatal medical intervention for the fetus is limited, and structural anomalies cannot be reversed during fetal development, primary prevention of congenital anomalies based on preconception care is critical at the population level. Identification of risk factors for congenital anomalies is fundamental for such primary prevention. Additional robust evidence and analytical methods are necessary to control for potential confounding factors.

## 12. Limitations

It is worth noting that the clinical studies conducted to date are beset by a number of characteristics that make them difficult to compare, such as small group sizes, heterogeneity of patient samples and insufficient sample sizes, which may cause errors and potential systematic bias, thus hindering comparative analysis of patients and control groups; also, different observation periods, study methods and rates of CHDs, as well as varying numbers of repeated CHDs, may also contribute to discrepancies in the final conclusions. Furthermore, definitions of maternal obesity vary widely, from pre-pregnancy BMI to weight gain during pregnancy, as is the case with the assessment of diabetes—fasting blood glucose, oral glucose tolerance test (OGTT), and glycated hemoglobin (HbA1c)—which gives rise to inconsistencies across the literature. In addition, many researchers fail to consider the variability in activity which can be influenced by cultural behaviors, air pollution, smoking exposure, alcohol consumption, medication use during pregnancy, and socioeconomic status in patients.

Furthermore, study populations may consist of heterogeneous ethnic groups with different cultural behaviors, diets and lifestyles. Additionally, many studies are based on heterogeneous clinical data, which also prevents in-depth comparative analysis or precise stratification and sensitivity analyses. Studies may be conducted in different laboratories at different times, using different research methods, which also limits the generalizability of the final results. Many mechanistic insights are drawn from animal studies, which may not fully capture the complexity of human physiology and maternal–fetal interactions. Additionally, most studies are short- to medium-term, with limited longitudinal follow-up into adolescence or adulthood, restricting the ability to fully capture intergenerational effects. Therefore, conclusions should be interpreted with caution. Importantly, clinical studies investigating the co-occurrence of CHDs in the offspring of mothers with diabetes and obesity are primarily cohort studies, which are burdened with a low level of evidence. Therefore, a systematic review based on randomized controlled trials is necessary to confirm the association between the coexistence of obesity and diabetes, their interplay and the development of CHDs.

## 13. Summary

This narrative review summarizes the current state of knowledge on the development of CHDs in patients with mothers affected by obesity or diabetes. The association between maternal diabetes or obesity and the development of CHD in offspring remains complex and multifaceted. The work analyses the mechanisms and cell signaling pathways through which maternal diabetes or obesity increases the risk of CHDs.

Maternal obesity and diabetes (pre-pregnancy and gestational diabetes) are important environmental factors that, through intrauterine metabolic disorders, affect the key molecular pathways of fetal heart development, significantly increasing the risk of CHDs. High levels of glucose (hyperglycemia), free fatty acids and inflammation in the mother’s body lead to increased oxidative stress, overproduction of ROS and DNA damage in the developing fetal cardiomyocytes, which disrupts the following signaling pathways: the TGF-\(\beta\) (Transforming Growth Factor-beta) pathway, the activation of which leads to increased cardiac fibrosis and cardiomyocyte hypertrophy, which disrupts the proper formation of the septa and valves; the Wnt/(beta)-ketamine pathway, the disruption of which in maternal diabetes can lead to abnormal Wnt signaling, which results in defects in the septum—ventricular septal defects (VSDs) and conotruncal defects (CTDs); the Hif1α pathway, disorders in which in maternal diabetes contribute to fetal CHDs; the insulin pathway (IGF/PI3K/AKT), where inappropriate regulation of the insulin pathway in response to fetal hyperinsulinemia (resulting from maternal diabetes) leads to growth disorders and functional immaturity of the heart cells; Notch signaling, which plays a role in the formation of coronary vessels; the renin–angiotensin–aldosterone system (RAAS), which is activated by obesity, resulting in the remodeling of the heart; Rho GTPase and actin cytoskeleton signaling, disturbances in which affect cell migration and cardiac development, leading to septal defects; inflammatory pathways (VCAM-1, E-selectin, and NOX), which are responsible for the creation of a pro-inflammatory environment (induced by obesity and diabetes) that activates adhesion molecules and increases oxidative stress, which damages heart structures.

Additionally, the development of the fetal heart in diabetes and obesity may be negatively affected by: (1) oxidative stress and apoptosis: excess glucose and lipids generate reactive oxygen species (ROS), which cause DNA damage and accelerated, abnormal cell death (apoptosis) during the critical period of organogenesis (the first trimester); (2) epigenetic disorders: intrauterine conditions (obesity/diabetes) alter the expression of genes important for heart development through DNA methylation and histone modifications, which permanently programs cardiac functions (fetal programming); (3) extracellular matrix remodeling (ECM) disorders: changes in the activity of matrix metalloproteinases (MMPs) disrupt the formation of the heart structure, leading to defects in valves and septa; 4) energy metabolism disorders: fetal cardiomyocytes exposed to diabetes show impaired glucose utilization, resulting in their immaturity and poorer ability to contract.

Studies conducted in Europe and America have demonstrated that maternal diabetes is a risk factor associated with nearly all subtypes of CHD in offspring, while obesity and overweight are associated with an increased risk for complex defects and outflow tract obstruction and a decreased risk for ventricular septal defects. The risk of CHDs in the fetus increased gradually with increasing maternal BMI.

In contrast, observational studies conducted in China showed an increase in CHDs in underweight mothers compared to mothers with overweight or obesity. The lack of correlation between maternal obesity and congenital heart defects was attributed to the difference in the prevalence of obesity between the West and the East. A small number of mothers with overweight or obesity participated in that research. This explains why the results were inconsistent with the results from other studies that were conducted in Europe or America. Subgroup analysis by study design showed that the significant association between maternal overweight and increased risk of infants with CHDs existed only in case–control studies, while the significant association between maternal obesity and increased risk of infants with CHDs existed in both cohort studies and case–control studies. Dose–response studies demonstrated that each 5 kg/m^2^ increase in maternal BMI is accompanied by a 7% increment in the risk of infants with CHDs. The risk of CHDs in offspring was demonstrated to be higher in women with PGDM than in those with GDM in the conducted studies. However, the prevalence of offspring CHDs correlates with increased or poorly controlled maternal blood glucose levels, measured by glycated hemoglobin (HbA1c).

Many inconsistent results of observational studies, systemic reviews and meta-analyses were retrieved from research work evaluating the association between gestational diabetes mellitus and obesity and CHDs in offspring. These studies also demonstrated great heterogeneity in their design, exposure definitions, outcome definitions, and choice of covariates. Taking into account that these conditions often occur in parallel, better understanding of the contribution of each factor to offspring risk for CHD in general and for specific CHD subgroups may not only be helpful in prevention, but may also provide cues to direct future research in unraveling the underlying molecular-level mechanisms through the use of modern genomic methods.

## 14. Conclusions

Six key conclusions emerge from this narrative review:

1. Maternal obesity is an independent risk factor for overall congenital heart defects in offspring.
2. PGDM partially mediates the association between maternal obesity and CHDs in offspring.
3. Disturbed metabolic status of the mother during pregnancy complicated by obesity and diabetes results in an increased incidence of complex forms of CHD in offspring.
4. The risk of CHDs is significantly higher among mothers with PGDM than in those with GDM.
5. Screening for PGDM and the adjustment of glucose levels play an important role in reducing the risk of fetal heart defects in obese women.
6. The mechanisms underlying the associations between maternal obesity and the risk of offspring CHDs need to be further investigated to provide individualized treatment plans for high-risk-populations.

## Figures and Tables

**Table 1:** The criteria applicable for the diagnosis of metabolic syndrome (MetS) in women according to the National Cholesterol Education Program—Adult Treatment Panel III. MetS is diagnosed when 3 of the following 5 criteria are met.

Cardiometabolic MetS Risk Factors
Abdominal obesity determined by the assessment of waist circumference ≥88 cm
Fasting glycemia ≥ 100 mg/dL (5.6 mmol/L) or treatment of type 2 diabetes
Arterial blood pressure ≥ 130/85 mm Hg or treatment of arterial hypertension
Elevated triglycerides ≥ 150 mg/dL (1.7 mmol/L) or treatment of dyslipidemia
Too low HDL level < 50 mg/dL (1.3 mmol/L) or treatment of dyslipidemia

**Table 2:** Criteria for MetS definition.

IDF Definition of MetS 2020 (Women)	WHO Definition of MetS 2020 (Women)	Current Study Modified Definition of MetS in the Second Trimester
Waist circumference ≥ 80 cm + at least two criteria from:	Any form of insulin resistance (FPG ≥ 5.6 mmol/L or IGT ≥ 7.8 mmol/L or DM) + at least two criteria from:	GDM (24/28th week of gravidity) according to IADPSG + at least two criteria from:
TAG ≥ 1.7 mmol/L (or therapy)	TAG ≥ 1.7 mmol/L (or therapy)	TAG ≥ 1.7 mmol/L *
HDL-C ≤ 1.3 mmol/L (or therapy)	HDL-C ≤ 1.0 mmol/L	HDL ≤ 1.3 mmol/L *
BP ≥ 130/85 mmHg (or therapy)	BP ≥ 140/90 mmHg (or therapy)	BP ≥ 130/85 mmHg *
FPG ≥ 5.6 mmol/L or DM	BMI ≥ 30 kg/m^2^ or waist/hip ratio ≥ 0.85	BMI before pregnancy ≥30 kg/m^2^

Abbreviations: * measured in the second trimester of gravidity. BMI: body mass index; BP: blood pressure; DM: diabetes mellitus; FPG: fasting plasma glucose; GDM: gestational diabetes mellitus; HDL: high-density lipoprotein; IADPSG: International Association Diabetes Pregnancy Study Group; IDF: International Diabetes Federation; MetS: metabolic syndrome; TAG: triacylglycerol; WHO: World Health Organization. This table was taken from the article of Bartáková et al. [49] distributed under the terms of the Creative Commons Attribution License (CC BY-NC-ND/4.0/).

**Table 3:** Common environmental exposures/teratogens and associated CHDs.

Teratogen	Commonly Associated CHDs
Assisted reproductive technology	Mild CHDs more common than severe: Ventricular septal defects Ventricular free wall thickening Pericardial effusion Tricuspid regurgitation Displaced apex
Maternal diabetes	Ventricular septal defects Atrioventricular septal defects Tetralogy of Fallot Transposition of the great arteries Truncus arteriosus Hypoplastic left heart Patent ductus arteriosus
Maternal lupus	Fetal heart block
Maternal phenylketonuria (PKU)	Ventricular septal defects Tetralogy of Fallot Patent ductus arteriosus Hypoplastic left heart syndrome
Rubella	Patent ductus arteriosus Ventricular septal defects Atrial septal defects Peripheral pulmonary stenosis
Alcohol	Ventricular septal defects Atrial septal defects Patent ductus arteriosus
Lithium	Ebstein’s anomaly (10%) Tricuspid atresia
Hyperthermia	Conotruncal and obstructive defects
Antiepileptics	Ventricular septal defects Atrial septal defects Tetralogy of Fallot Pulmonary valve atresia Hypoplastic right heart
Vitamin A	Pulmonary stenosis and outflow tract abnormalities
Paroxetine	Ventricular septal defects Atrial septal defects Outflow tract obstructions
NSAIDs, such as Ibuprofen	Transposition of the great arteries Ventricular septal defects Bicuspid aortic valve

This table has been taken from the article of Rachamadugu et al. [11] distributed under the terms of the Creative Commons Attribution License (CC BY-NC-ND/4.0/).

**Table 4:** Common syndromes associated with congenital heart defects (CHDs).

**Chromosomal aneuploidies**				
**Syndrome**	**Gene**	**Locus**	**Cardiac defect**	**% CHD**
Down	Unknown	Trisomy 21	AVSDs, ASDs, VSDs, PDA, ToF	50
Turner	Unknown	45, X (monosomy X)	CoA, BAV, AS, HLHS	30
**CNVs**				
**Syndrome**	**Gene**	**Locus**	**Cardiac defect**	**% CHD**
22q11.2 deletion DiGeorge	*TBX1*	22q11.2 deletion	ToF, PTA, VSDs, ASDs, IAA (conotruncal defects)	74–85
1p36 deletion	Unknown 1p36 deletion	1p36 deletion	PDA, VSDs, ASDs, BAV, Ebsteinʹs anomaly	70
Wiliams–Beuren	*ELN*	7q11.23	SVAS, PPS, VSDs, ASDs	80
Jacobsen	*ETS1* *FLI1*	11q terminal deletion	HLHS, AS, VSDs, CoA, Shoneʹs complex	56
**Single-gene variation**				
**Syndrome**	**Gene**	**Locus**	**Cardiac defect**	**% CHD**
Alagille	*JAG1*	20p12.2	PPS, ToF, PA	>90
Char	*TFAP2B*	6p12.3	PDA, VSDs	58
CHARGE	*CHD7*	8q12	ToF, PDA, DORV, AVSDs, VSDs	75–85
Costello	*HRAS*	11p15.5	PS, ASDs, VSDs, HCM	44–52
Elis–van Creveld	*EVC*	4p 16.2	Common atrium	60
Holt–Oram	*TBX5*	12q24.1	VSDs, ASDs, AVSDs	50
Kabuki	*KMT2D*	12Q13	CoA, BAV, VSDs, ToF, TGA, HLHS	50
	*KDM6A*	Xp11.3		
Noonan	*PTPN11*	12q24.13	ASDs, VSDs, AVSDs, PAF, ToF, HCM, PDA, dysplastic PVS	75
	*SOS1*	2p22.1		
	*RAF1*	3p25.2		
	*KRAS*	12p12.1		
	*NRAS*	1p13.2		
	*RIT1*	1q22		
	*SHOC2*	10q25.2		
	*SOS2*	14q21.3		
	*BRAF*	7q34		

Abbreviations: CNV: copy number variation; CHD: congenital heart disease; ASD: atrial septal defect; VSD: ventricular septal defect; PDA: patent ductus arteriosus; ToF: tetralogy of Fallot; CoA: coarctation of the aorta; AVSD: atrioventricular septal defect; PA: pulmonary atresia; DORV: double-outlet right ventricle; BAV: bicuspid aortic valve; HLHS: hypoplastic left heart syndrome; AS: aortic stenosis. This table has been taken from the article of Yasuhara and Garg [10] distributed under the terms of the Creative Commons Attribution License (CC BY-NC-ND/4.0/).

**Table 5:** Genes associated with non-syndromic congenital heart defects (selected).

**Transcription factors**	
**Gene**	**Cardiovascular malformation**
*CITED2*	ASD, VSD
*GATA4*	ASD, VSD, AVSD, PS, ToF
*GATA5*	ASD, VSD, DORV, ToF, BAV
*GATA6*	PTA, ToF
*HAND1*	AVSD, DORV, HLHS, ASD, VSD
*HAND2*	ToF, LVNC, VSD
*JARID2*	Left-sided lesions
*MED13L*	TGA
*NR2F2*	AVSD, AS, CoA, VSD, HLHS, ToF
*NKX2-5*	ASD, atrioventricular conduction delay, ToF, HLHS, VSD
*NKX2-6*	PTA
*TBX1*	DORV, ToF, IAA, PTA, VSD
*TBX5*	AVSD, ToF, BAV, CoA, ASD, VSD
*TBX20*	ASD, VSD, MS, DCM
*MEF2C*	DORV
*NFATC1*	TA, AVSD
*ZFPM2/FOG2*	ToF, DORV
**Cell signaling and adhesion proteins**	
**Gene**	**Cardiovascular malformation**
*ACVR1/ALK2*	AVSD
*CFC1*	TGA, DORV
*CRELD1*	ASD, AVSD
*FOXH1*	ToF, TGA, VSD
*GDF1*	ASD, DORV, TGA, ToF
*GJA1*	HLHS, VSD, PA
*HEY2*	AVSD
*JAG1*	ToF, PS
*NODAL*	TGA, DORV, ToF, VSD
*NOTCH1*	BAV, AS, HLHS, ToF, PS, ASD, VSD, CoA, DORV
*PDGFRA*	TAPVR
*SMAD6*	BAV, CoA, AS
*TAB2*	BAV, AS, ToF
*VEGFA*	ToF, PDA, AS, BAV, CoA, IAA, VSD
**Structural proteins**	
**Gene**	**Cardiovascular malformation**
*ACTC1*	ASD, HCM, DCM, LVNC
*DCHS1*	MVP
*ELN*	SVAS
*MYH6*	ASD, HCM, DCM
*MYH7*	LVNC, HCM, DCM, Ebsteinʹs anomaly
*MYH11*	PDA, TAA

Abbreviations: AS: aortic stenosis; ASD: atrial septal defect; AVSD: atrioventricular septal defect; BAV: bicuspid aortic valve; CoA: coarctation of the aorta; DCM: dilated cardiomyopathy; DORV: double-outlet right ventricle; HCM: hypertrophic cardiomyopathy; HLHS: hypoplastic left heart syndrome; IAA: interrupted aortic arch; LVNC: left ventricular noncompaction; MS: mitral stenosis; MVP: mitral valve prolapse; PA: pulmonary atresia; PDA: patent ductus arteriosus; PS: pulmonary valve stenosis; PTA: persistent truncus arteriosus; SVAS: supravalvular aortic stenosis; TA: tricuspid atresia; TAA: thoracic aortic aneurysm; TAPVR: total anomalous pulmonary venous return; TGA: transposition of the great arteries; ToF: tetralogy of Fallot; VSD: ventricular septal defect. This table has been taken from the article of Yasuhara and Garg [10] distributed under the terms of the Creative Commons Attribution License (CC BY-NC-ND/4.0/).

**Table 6:** Congenital heart defects (CHDs) and associated miRNAs.

Pathology	Involved miRNAs	Target Genes
ToF	miR-27b, miR-421, miR-1275, miR-122, miR-1201, miR-22, miR-222, miR-375, miR-138, miR-421, miR-1, miR-206, miR-940, hsa-miR-148a, hsa-miR-221-3p, hsa-miR-218-5p, hsa-miR-873-5p, miR-19, let-7e-5p, miR-10a-5p, miR-181c, miR-940, miR-181, miR-130, miR-146b-5p, miR-29c, miR-720, miR-424, miR-660, miR-708, miR-363, miR-337-5p, miR-155, miR-154	*SOX4*, *BCL2L11*, *TBX5*, *CDK9*, *FN1*, *MAPK1*
HLHS	miR-30, miR-100, miR-378, miR-99a, miR-145a, miR-208, miR-204	*QKI*, *FOG2*, *CDK6*, *SOX11*, *BAZ2A*
ASD	miR-20b-5p, hsa-miR-19b, hsa-miR-375, hsa-miR-29c, miR-139-5p, miR-196-a2, miR-9, miR-30a, hsa-let-7a, hsa-let-7b, hsa-miR-486, miR-29, miR-143/145, miR-17-92, miR-106b-25, miR-503/424	*ACTC1*, *TBX5*, *PTEN*, *VEGFR-1*
VSD	miR-1/2, miR-1/1, miR-181c, miR-92, let-7e-5p, miR-155-5p, miR-222-3p, miR-379-5p, miR-409-3p, miR-433, miR-487b, miR-498	*GJA1*, *SOX9*, *BMPR2*,
BAV	miR-26a, miR-95, miR-30b, miR-141	*BMP2*, *ALPL*, *SMAD1*, *SMAD3*
TGA	has-let-7e, miR-16, miR-25, miR-18a, miR-93, miR-106a, miR-451, miR-486-3p, miR-486-5p	*ATM*, *PTEN*, *BCL11A*, *FOXO1*, *MMP19*, *IGF1*, *HAT1*, *SMAD1*
Down syndrome	miR-99a, has-let-7c, miR-125b2, miR-155, miR-802	*IL10*, *NOX4*, *RUNX3*, *CYP24A1*
DiGeorge syndrome	miR-23, miR-363, let-7g, miR-361-5p, miR-324-5p, miR-194, miR-720, miR-150, miR-15b-3p, miR-185	*SOX17*, *AFP*, *G3BP2*

This table has been taken from the article of Rodríguez-Pérez et al. [187] distributed under the terms of the Creative Commons Attribution License (CC BY-NC-ND/4.0/).

**Table 7:** Genetic testing indications.

Test	Definition	Associated CHD Conditions
Fluorescence in situ hybridization (FISH)	Detection of suspected deletion or duplication syndromes of specific DNA regions	Trisomy 21, 18, and 13 Turner syndrome Williams syndrome 22q11.2 deletion syndrome
Multiplex ligation-dependent probe amplification	Assessment of known microdeletion/duplication syndromes; detection of CNVs	1p36 deletion syndrome Williams syndrome 22q11.2 deletion syndrome
Chromosomal microarray analysis (CMA)	Evaluation of patients with multiple congenital anomalies to identify underlying chromosomal abnormalities or CNVs affecting critical periods of cardiac development	HLHS CHARGE syndrome Jacobsen syndrome Alagille syndrome 22q11.2 deletion syndrome
Whole-exome sequencing (WES)	Selective sequencing of protein-coding regions, accounting for approximately 1% of the genome; precise identification of single-nucleotide variants, CNVs, insertions, and microdeletions	ToF ASD PDA HLHS Aortic valve stenosis
Whole-genome sequencing (WGS)	Sequencing of the entire genome, including non-coding and protein-encoding regions. Identification of SNVs, insertions, microdeletions, and structural variants	ToF Coarctation of the aorta (CoA) CHARGE syndrome Alagille syndrome
Comparative genomic hybridization arrays	Assessment of unbalanced large CNV changes and genomic rearrangements	22q11.2 deletion syndrome Wolf–Hirschhorn syndrome Miller–Dieker syndrome HLHS ToF VSD CoA

Abbreviations: ASD: atrial septal defect; CHARGE: coloboma, heart defect, atresia choanae, growth retardation, genital and ear abnormalities; CHD: congenital heart disease; CNV: copy number variation; HLHS: hypoplastic left heart syndrome; PDA: patent ductus arteriosus; SNV: single-nucleotide variant; ToF: tetralogy of Fallot; VSD: ventricular septal defect. This table has been taken from the article of Chhatwal et al. [12] distributed under the terms of the Creative Commons Attribution License (CC BY-NC-ND/4.0/).

**Table 8:** Classification of pregnant diabetic patients according to White.

Class A	Appropriate diet is sufficient, any age of onset and duration of diabetes
Class B	Onset of diabetes at the age of ≥20 years or duration of diabetes <10 years
Class C	Onset of diabetes at the age of 10–19 years or duration of diabetes 10–19 years
Class D	Onset of diabetes at the age of <10 years or duration of diabetes >20 years or non-proliferative retinopathy or arterial hypertension
Class R	Proliferative retinopathy or vitreous hemorrhages present
Class F	Overt nephropathy present
Class RF	R+F criterion
Class H	Clinically overt ischemic heart disease
Class T	Past kidney transplantation

**Table 9:** Criteria for diagnosis of gestational diabetes mellitus (GDM) and diabetes in pregnancy—based on Polish Diabetes Association: Clinical recommendations for management in people with diabetes 2025. Position of the Polish Diabetes Association [218].

Gestational Diabetes Mellitus (One of Three Criteria)	Diabetes in Pregnancy (One of Three Criteria)
Fasting plasma glucose: 92–125 mg/dL	Fasting plasma glucose: ≥126 mg/dL
1 h plasma glucose after loading of 75 g glucose ≥180 mg/dL	2 h plasma glucose after loading of 75 g glucose: ≥200 mg/dL
2 h plasma glucose after loading of 75 g glucose: 153–199 mg/dL	Random plasma glucose ≥200 mg/dL and clinical symptoms of hyperglycemia

**Table 10:** Common genetic variant association loci for T2DM.

SNP	Chr	GENES
rs17106184	1	*FAF1*
rs10923931	1	*NOTCH 2*, *ADAM30*
rs340874	1	*PROX1*
rs780094	2	*GKKR*
rs7578597	2	*THADA*
rs243021	2	*BCL11A*
rs7560163	2	*RBM43*, *RND3*
rs7593730	2	*ITGB6*, *RBSMS1*
rs3923113	2	*GRB14*
rs7578326	2	*IRS1*
rs1801282	3	*PPARG*
rs7612463	3	*UBE2E2*
rs831571	3	*PSMD6*
rs4607103	3	*ADAMTS9*
rs11717195	3	*ADCY5*
rs4402960	3	*IGF2BP2*
rs16861329	3	*ST6GAL1*
rs6808574	3	*LPP*
rs6815464	4	*MAEA*
rs1801214	4	*WFS1*
rs6813195	4	*TMEM154*
rs702634	5	*ARL15*
rs4457053	5	*ZBED3*
rs9502570	6	*RREB1*, *SSR1*
rs7754840	6	*CDKAL1*
rs3132524	6	*TCF19*, *POU5F1*
rs9470794	6	*ZFAND3*
rs1535500	6	*KCNK16*
rs1048886	6	*C6orf57*
rs2191349	7	*DGKB*, *TMEM195*
rs864745	7	*JAZF1*
rs4607517	7	*GKK*
rs6467136	7	*PAX4*, *GCC1*
rs791595	7	*MIR129*, *LEP*
rs972283	7	*KLF14*
rs515071	8	*ANK1*
rs896854	8	*TP53INP1*
rs13266634	8	*SLC30A8*
rs7041847	9	*GLIS3*
rs17584499	9	*PTPRD*
rs10811661	9	*CDKN2A*, *CDKN2B*
rs13292136	9	*CHCHD9*
rs11787792	9	*GPSM1*
rs12779790	10	*CDC123*, *CAMK1D*
rs1802295	10	*VPS26A*
rs12571751	10	*ZMIZ1*
rs1111875	10	*HHEX*
rs7903146	10	*TCF7L2*
rs10886471	10	*GRK5*
rs3842770	11	*INS-IGF2*
rs2237892	11	*KCNQ1*
rs5215	11	*KCNJ11*
rs1552224	11	*CENTD2*
rs1387153	11	*MTNR1B*
rs1531343	12	*HMGA2*
rs7961581	12	*LGR5*, *TSPAN8*
rs7957197	12	*HNF1A*
rs1727313	12	*MPHOSPH9*
rs9552911	13	*SGC*, *SACS*
rs1359790	13	*SPRY2*
rs7403531	15	*RASGRP1*
rs7172432	15	*C2CD4B*, *C2CD4A*
rs7178572	15	*HMG20A*
rs11634397	15	*ZFAND6*
rs2028299	15	*AP3S2*
rs8042680	15	*PRC1*
rs8050136	16	*FTO*
rs391300	17	*SRR*
rs312457	17	*SLC16A13*
rs4430796	17	*HNF1B*
rs8090011	18	*LAMA1*
rs12970134	18	*MC4R*
rs3786897	19	*PEPD*
rs4812829	20	*HNF4A*
rs5945326	23	*DUSP9*

Abbreviations: Chr: chromosome number; SNP: variant rsID; T2DM: type 2 diabetes mellitus. This table has been adapted with some modifications from the article of Kwak and Park [334] distributed under the terms of the Creative Commons Attribution License (CC BY-NC-ND/4.0).

**Table 11:** Pooled RR and 95% confidence intervals for associations between PGDM and GDM and any type of CHD.

Outcome	Number of Events	Pre-Gestational Diabetes				Gestational Diabetes			
		Number of Studies	Pooled RR (95% CI)	I^2^ (%)	*p* Value	Number of Studies	Pooled RR (95% CI)	I^2^ (%)	*p* Value
**Heterotaxia**	1.098	4	8.78 (6.66 to 11.56)	0.0	0.423	2	5.70 (1.09 to 29.92)	85.7	0.008
**Conotruncal defects**	5.495	4	3.76 (2.58 to 5.48)	68.3	0.024	--			
Truncus arteriosus	435	3	12.16 (7.52 to 19.68)	0.0	0.866	2	1.77 (0.80 to 3.92)	40.2	0.196
Transposition of great vessels	6.700	9	3.25 (2.54 to 4.15)	15.9	0.301	2	1.29 (0.99 to 1.67)	61.2	0.109
Tetralogy of Fallot	5.360	6	3.46 (2.27 to 5.28)	64.4	0.015	2	1.41 (1.20 to 1.66)	0.0	0.600
**APVR**	1.239	4	3.47 (2.13 to 5.64)	0.0	0.684	2	1.42 (0.79 to 2.56)	53.3	0.117
**LVOT defects**	6.672	7	3.46 (2.59 to 4.62)	37.8	0.140	4	1.67 (1.15 to 2.41)	50.0	0.112
Coarctation of aorta	6.606	5	3.35 (2.25 to 4.99)	61.4	0.035	2	1.50 (1.23 to 1.83)	35.4	0.213
Hypoplastic left heart	2.319	4	2.23 (1.07 to 4.64)	64.0	0.040	2	1.23 (0.54 to 2.82)	81.7	0.019
**RVOT defects**	6.163	7	3.41 (2.65 to 4.38)	20.9	0.270	3	1.25 (1.03 to 1.53)	0.0	0.739
Pulmonary artery anomalies	17.215	3	2.81 (2.48 to 3.18)	0.0	0.865	2	1.02 (0.36 to 2.87)	71.6	0.060
Pulmonary valve stenosis	7.273	5	2.51 (1.51 to 4.17)	76.2	0.002	2	1.30 (0.96 to 1.76)	64.5	0.093
**Septal defects**	12.368	2	3.23 (2.20 to 4.74)	86.2	0.007	--			
AVSD	5.126	6	3.94 (2.95 to 5.26)	40.0	0.139	3	1.02 (0.83 to 1.24)	0.0	0.751
VSD	64.844	10	3.10 (2.32 to 4.16)	90.2	<0.001	2	1.31 (1.24 to 1.38)	0.0	0.960
ASD	91.683	7	3.12 (2.42 to 4.02)	81.9	<0.001	2	1.45 (1.40 to 1.50)	0.0	0.426
VSD+ASD	1.089	2	6.36 (4.38 to 9.24)	0.0	0.527	--			
**Single ventricle**	1.228	4	5.91 (2.43 to 14.38)	80.2	0.002	2	1.14 (0.77 to 1.69)	0.0	0.851

Abbreviations: AVPR: anomalous pulmonary venous return; ASD: atrioventricular septal defect; CHD: congenital heart defect; CI: confidence interval; LVOT: left ventricular outflow tract; RR: relative risk; RVOT: right ventricular septal defect. This table has been taken from the article of Zhang et al. [362] distributed under the terms and conditions of the Creative Commons Attribution License (CC BY-NC-ND/4.0).

**Table 12:** WHO obesity classification based on BMI (own modification).

BMI (kg/m^2^)	WHO Classification
<18.5	Underweight
18.5–24.9	Normal weight
25.0–29.9	Overweight
30.0–34.9	Class I obesity
35.0–39.9	Class II obesity
≥40.0	Class III obesity (giant morbid obesity)

**Table 13:** Overview of association between congenital heart defect (CHD) subtypes and maternal exposure to metabolic syndrome (MetS) diseases.

	Obesity	PGDM	DM1	DM2	GDM	Hypertension	PE (Early-Onset)	Obesity + GDM
Heterotaxia	2 ↓	3(2) ↑	1 ↑	1(1) ↑	2 ↑	1 ↑ 1 ↓	2(1) ↑ 1 ↓	1 ↑
UVH	2 ↑	2(1) ↑	NA	NA	1 ↑	1 ↑ 1 ↓	1(1) ↑	1 ↓
Conotruncal defects	4(2) ↑ 1 ↓	3(2) ↑	1(1) ↑	1(1) ↑	1 ↑	2(1) ↑	1(1) ↑	1 ↑
Common truncus	1 ↑	3(2) ↑	NA	NA	1(1) ↑	2(1) ↑	1(1) ↑	NA
TGA	8(2) ↑ 3 ↓	6(3) ↑	NA	NA	2 ↑	2 ↓	2 ↓	1 ↓
ToF	7(3) ↑ 4 ↓	5(4) ↑	NA	NA	2(1) ↑	2(1) ↑	1 ↑ 1 ↓	1(1) ↑
DORV	1 ↑	1(1) ↑	NA	NA	NA	NA	1(1) ↑	NA
Aortic arch defects	1(1) ↑	NA	NA	NA	NA	NA	NA	NA
AVSD	6(2) ↑	6(5) ↑	1(1) ↑	1(1) ↑	3(1) ↑ 1 ↓	3(2) ↑	3(3) ↑	1 ↑
TAPVR	1 ↑ 1 ↓	4(2) ↑	NA	NA	1(1) ↑ 1 ↓	1 ↓	1(1) ↑	1 ↑
LVOT	2(1) ↑ 2 ↓	4(4) ↑	1(1) ↑	1(1) ↑	2(1) ↑	1 ↑	1 ↓	1(1) ↑
HLHS	5(2) ↑ 1 ↓	3(1) ↑	NA	NA	1 ↑ 1 ↓	1 ↑ 1 ↓	1 ↑ 1 ↓	1(1) ↑
CoA	5 ↑ 1 ↓	5(3) ↑	NA	NA	2(1) ↑	1(1) ↑ 1 ↓	2 ↑	1 ↑
Aortic stenosis	2(1) ↑ 1 ↓	4(3) ↑	NA	NA	1(1) ↑ 1 ↓	1 ↑ 1 ↓	NA	1 ↑
RVOT	3(2) ↑ 1 ↓	4(3) ↑	1↑	1↑	2(1) ↑	1(1) ↑	1(1) ↑	1(1) ↑
Pulmonary stenosis	4(2) ↑	3(2) ↑	NA	NA	2(1) ↑	1(1) ↑	NA	1(1) ↑
Ebstein’s anomaly	1 ↑	1(1) ↑	NA	NA	1 ↑	2 ↑	1 ↑	NA
Septal defects	3(1) ↑	2(2) ↑	NA	NA	1(1) ↑	NA	1(1) ↑	1(1) ↑
VSD	10(3) ↑	7(5) ↑	1(1) ↑	1(1) ↑	2(1) ↑	3(3) ↑	3(3) ↑	1 ↑
ASD	9(6) ↑	5(3) ↑ 1 ↓	1(1) ↑	1(1) ↑	2(1) ↑	2(2) ↑1 ↓	2(2) ↑	1(1) ↑

Numbers reflect the numbers of studies examining specific combinations of CHD subtypes and exposures. Numbers of significant associations are given in bold and in brackets. Arrows reflect positive (↑) and negative (↓) directions of the associations. Abbreviations: ASD: atrial septal defect; AVSD: atrioventricular septal defect; CoA: coarctation of the aorta; DM1: diabetes type 1; DM2: diabetes type 2; DORV: double-outlet right ventricle; GDM: gestational diabetes; HLHS: hypoplastic left heart syndrome; LVOT: left ventricular outflow tract defects; PE: preeclampsia; PGDM: pregestational diabetes; RVOT: right ventricular outflow tract defects; TAPVR: total anomalous pulmonary venous return; TGA: transposition of the great arteries; ToF: Tetralogy of Fallot; UVH: univentricular heart; VSD: ventricular septal defect. This table has been taken from the article of Hedermann et al. [21] distributed under the terms and conditions of the Creative Commons Attribution License (CC BY-NC-ND/4.0).

## Data Availability

No new data were created or analyzed in this study. Data sharing is not applicable to this article.

## Abbreviations

95% CI	95% confidence interval
aOR	Adjusted odds ratio
ARTs	Assisted reproductive techniques
AS	Aortic valve stenosis
ASD	Atrial septal defect
AVSD	Atrioventricular septal defect
BMI	Body mass index
CHD	Congenital heart disease / defect
CI	Confidence interval
CoA	Aortic coarctation
CTD	Conotruncal defect
CVD	Cardiovascular disease
DCM	Diabetic cardiomyopathy
DM	Diabetes mellitus
DNV	De novo variant
DORV	Double-outlet right ventricle
EUROCAT	European Surveillance of Congenital Anomalies
EWAS	Epigenome-wide association study
FHF	First heart field
GDM	Gestational diabetes mellitus
GH	Growth hormone
GLUT-1	Glucose transporter-1
GLUT-4	Glucose transporter-4
GnRH	Gonadotrophin releasing hormone
GWAS	Genome-wide association study
HbA1C	Glycosylated hemoglobin A
HDL	High-density lipoprotein
HTX	Heterotaxy
HLHS	Hypoplastic left heart syndrome
ICD	International Classification of Diseases
IGF-1	Insulin-like growth factor 1
IR	Insulin resistance
LADA	Latent autoimmune diabetes in adults
LDL	Low-density lipoprotein
LVOTO	Left ventricular outflow tract obstruction
matDM	Maternal diabetes mellitus
MODY	Maturity onset diabetes of the young
MR	Mendelian randomization
MetS	Metabolic syndrome
ncRNA	Non-coding RNA
NGS	Next-generation sequencing
NF-κB	Nuclear factor kappa B cell
OFT	Cardiac outflow tract
OGTT	Oral glucose tolerance test
OR	Odds ratio
OS	Oxidative stress
PA	Pulmonary artery
PDA	Patent ductus arteriosus
PE	Preeclampsia
PGDM	Pregestational diabetes mellitus
PM_2.5_	Fine particulate matter
PRR	Prevalence rate ratio
PS	Pulmonary valve stenosis
PTA	Persistent truncus arteriosus
RNA	Ribonucleic acid
ROS	Reactive oxygen species
RVOTO	Right ventricular outflow tract obstruction
SD	Septal defect
SHF	Second heart field
T1DM	Type 1 diabetes mellitus
T2DM	Type 2 diabetes mellitus
TA	Truncus arteriosus
TGA	Transposition of the great arteries
TNF-α	Tumor necrosis factor alpha
ToF	Tetralogy of Fallot
UVH	Univentricular heart
VSD	Ventricular septal defect
WHO	World Health Organization
